# Factors influencing respectful perinatal care among healthcare professionals in low-and middle-resource countries: a systematic review

**DOI:** 10.1186/s12884-024-06625-6

**Published:** 2024-06-24

**Authors:** Petronellah Lunda, Catharina Susanna Minnie, Welma Lubbe

**Affiliations:** 1 School of Nursing, North-West University, NuMIQ Research Focus Area, Potchefstroom, South Africa; 2 School of Nursing, University of the Western Cape, Bellville, Cape Town, South Africa

**Keywords:** Doctors, Maternity care, Midwives, Nurses, Obstetrician, Perceptions, Perinatal care, Respectful care

## Abstract

**Background:**

This review aimed to provide healthcare professionals with a scientific summary of best available research evidence on factors influencing respectful perinatal care. The review question was ‘What were the perceptions of midwives and doctors on factors that influence respectful perinatal care?’

**Methods:**

A detailed search was done on electronic databases: EBSCOhost: Medline, OAlster, Scopus, SciELO, Science Direct, PubMed, Psych INFO, and SocINDEX. The databases were searched for available literature using a predetermined search strategy. Reference lists of included studies were analysed to identify studies missing from databases. The phenomenon of interest was factors influencing maternity care practices according to midwives and doctors. Pre-determined inclusion and exclusion criteria were used during selection of potential studies. In total, 13 studies were included in the data analysis and synthesis. Three themes were identified and a total of nine sub-themes.

**Results:**

Studies conducted in various settings were included in the study. Various factors influencing respectful perinatal care were identified. During data synthesis three themes emerged namely healthcare institution, healthcare professional and women-related factors. Alongside the themes were sub-themes human resources, medical supplies, norms and practices, physical infrastructure, healthcare professional competencies and attributes, women’s knowledge, and preferences. The three factors influence the provision of respectful perinatal care; addressing them might improve the provision of this care.

**Conclusion:**

Addressing factors that influence respectful perinatal care is vital towards the prevention of compromised patient care during the perinatal period as these factors have the potential to accelerate or hinder provision of respectful care.

**Supplementary Information:**

The online version contains supplementary material available at 10.1186/s12884-024-06625-6.

## Background

Respectful perinatal care (RPC) is integral to providing quality intrapartum care to women within the health care system [1]. Traditionally, women have given birth at home, surrounded by familiar caregivers within the community. However, the last quarter of the 20th century saw an increase in facility births as well as advances in medical expertise and interventions during these births [2]. Facility births expose women to standard hospital practices that do not have a scientific basis [3]. Additionally, facility births expose women to unfamiliar environments with strangers which can be terrifying for women; thus, healthcare professionals (HCPs) have a responsibility to provide a safe and secure environment [4]. Incidences of disrespect and abuse (D&A) during childbirth have been reported during the perinatal period [5–8]. D&A permeates the entire maternity culture propagated by both men and women [9]. Sadler et al. [10] identify non-evidence-based practice (non-EBP) as a form of D&A that has attracted international attention. The World Health Organization [11] highlights that HCPs often fail to recognise inexpensive, non-clinical approaches such as labour companionship, effective communication, and respectful care. These approaches are not only essential components of quality care but are also prioritised in many maternity settings to ensure positive maternal and neonatal outcomes. Byrom and Downe [12] urge HCPs to use evidence-based approaches to limit D&A.

Miller et al. [13] highlight two extreme forms of care on the continuum of maternity care: “too little, too late (TLTL)” associated with low and middle-income countries and “too much, too soon (TMTS)” mostly practised in high-income countries. TLTL pertains to the unavailability of resources, as well as substandard and inaccessible care, that contribute to poor outcomes. TLTL and TMTS both evoke feelings of one ‘being’ a product waiting to be ‘processed’ in a production line due to a lack of compassionate care from HCPs [14, 15].

Van Teijlingen [16] contrasts a medical and social model of care. A medical model is associated with obstetric practice and is technocratic. It views pregnancy as a risk that must be minimised and the woman as a passive recipient of care. The author [16] further states that the social model associated with midwifery practice is woman-centred. It perceives childbirth as a normal process that does not need routine interventions.

Organisational factors contributing to poor maternal healthcare provision are complex [17]. These include lack of support from superiors, shortage of healthcare professionals (HCPs), inadequate resources, poor infrastructure, low salaries, high caseloads, and adverse working conditions [18, 19]. Human Rights Watch (HRW) [8] identifies a lack of accountability by HCPs and administrators as another factor. These factors contribute to poor HCP behaviour often associated with work-related stress, highlighting the synchrony between improving the healthcare environment and correcting HCP abusive behaviours [17]. In low-resource settings such as South Africa, HCPs working in the public sector often experience these adverse working conditions [7, 20].

The high incidences of D&A in Brazil led to the promulgation of legislation that permits the presence of women’s rights movements within healthcare institutions to tackle issues of D&A [21]. A study by Pickles [22], “Eliminating abusive care: a criminal law response to obstetric violence in South Africa”, concluded that there is a need for a discourse on ways of eliminating D&A as well as the feasibility of “legislation prohibiting obstetric violence.”

Disrespect and abuse contradict what healthcare services should represent, namely accessibility, efficiency, and upholding human rights [5]. Oosthuizen et al. [23] concluded that interventions to promote RPC corresponding with support for HCPs and accountability by local maternity authorities is vital at all levels of care. Maternal healthcare services should not only focus on preventing maternal mortality and morbidity but also facilitate positive experiences for women who utilise the services [5].

### Purpose

The purpose of this review was to explore and synthesise evidence on factors influencing respectful perinatal care among midwives and doctors. The qualitative synthesis of studies focusing on the perceptions of doctors and mid?wives generated comprehensive scientific evidence on factors influencing respectful care.

## Research methods

A systematic review methodology underpinned by an exploratory, descriptive design was used to answer the review question. A systematic review uses logical, structured, and connected steps [24]. The steps of the systematic review followed the evidence analysis process of the Academy of Nutrition and Dietetics (AND) [25]: (1) Preparation of the review question and search plan, (2) searching for evidence, (3) critical appraisal of the studies, (4) data analysis and summarising the findings and (5) writing the conclusion.

### Formulation of the review question and search strategy

The review question aided the search for relevant studies [25]. In this study, the Sample Phenomenon of Interest Design Evaluation and Research method (SPIDER) assisted in formulating the review question [26] (See Table 1). The review question was ‘What were the perceptions of midwives and doctors on factors influencing respectful perinatal care?’

**Table 1:** Elements of the review question according to SPIDER (Cooke et al.)

Elements of the SPIDER	Elements as applied in the current study	Search words and synonyms of the elements
S- Sample	Doctors and midwives	Doctors, obstetricians, physicians, midwives, nurses
PI - Phenomenon of Interest	Respectful care	Respectful care, humanising maternity care, women-centred or woman-centred
D - Design	Qualitative and mixed-method research	Qualitative and mixed-method research
E - Evaluation	Perceptions of factors influencing respectful care	Perceptions, views, opinions, and experiences of factors influencing respectful care
R - Research method	Semi-structured interviews, in-depth or focus group interviews, case studies	Semi-structured, in-depth or focus group interviews, case studies

The first author and the faculty librarian were involved in refining the search strategy for identifying evidence from the databases using search words [25]. Elements of SPIDER and their synonyms were used as search words in the search string to avoid missing studies [27].

(Midwives or midwife) OR (nurse or nurses) AND.

(Obstetrician) OR (physicians or doctors) AND.

(Perception OR opinion OR attitude) AND.

(Healthcare professional) AND.

(Respectful perinatal care) OR (respectful care) OR (humanising delivery).

### Executing the search

The literature included both electronic and manual searches to ensure an exhaustive exploration. A PRISMA flow diagram was used to report the search process [28].

#### Electronic and manual search

The electronic databases and search included the following: EBSCO host databases, Science Direct, Cumulative Index to Nursing and Allied Health Literature (CINAHL), Cochrane collaboration database, PubMed, Web of Science, and Psych Articles. Nexus was searched to identify dissertations that have not been published yet.

Studies identified from databases and PubMed (most studies) were imported into the Evidence for Policy and Practice Information (EPPI) Reviewer 4 through Research Information Systems (RIS) formatted files; the EPPI Reviewer 4 also assisted in removing duplicates [29, 30]. The EPPI Reviewer 4 is a web-based software programme that can manage and analyse large volumes of data in literature, and systematic reviews. Thereafter a manual search of reference lists of studies identified from the electronic search was done to find any relevant studies that could have been missed during the search [31]. All identified studies were subsequently assessed on eligibility.

#### Assessment on eligibility

Once duplicates were removed, the next step was screening the titles and abstracts to exclude studies that were irrelevant to the research topic. Subsequently, full texts of the remaining studies were screened to determine whether they met the inclusion criteria (See Table 2). Only studies meeting the inclusion criteria were included in the critical appraisal.

**Table 2:** Inclusion and exclusion criteria

Inclusion criteria	Exclusion criteria
• Studies published from 2010 (since the research of respectful maternity care started after 2010). • Studies published in English or with English abstracts enabled the researcher to decide on the document’s relevance. • Studies using qualitative and mixed methods designs with the following data collection methods: focus group interviews, semi-structured interviews and observation that used qualitative methods for data analysis. • Surveys with open-ended responses.	• Non-primary research • Non-research reports • Studies about aspects of maternity care other than perinatal care. • Studies that focused on other healthcare professionals other than doctors and midwives.

### Critical appraisal of selected studies

To evaluate the quality of the studies, two researchers independently appraised the studies on quality and then compared the outcomes for consensus. The third member co-checked the decisions. The Critical Appraisal Skills Programme (CASP) tool for qualitative studies [32] and the Johns Hopkins Nursing Evidence-Based Practice (JHNEBP) appraisal tool [33] were used. The specific CASP tool was used for qualitative studies as it provides a methodological rating for the quality of primary qualitative studies [34].

The problem statement, the purpose of the study, literature review, sample size, control group, data collection methods, instrument reliability and the response rate to the survey or questionnaire of the individual studies were appraised. The agreed cut-off score for inclusion was 7/10 for the CASP tool [25] and ‘good quality’ for the JHNEBP [34]. Primary studies that scored high and those of good quality, were comprehensive with a detailed methodology, used multiple sources to support the data, researchers acknowledged and avoided their own biases, the focus was on participants’ responses, and the data were interpreted meaningfully with existing literature. Low-grade studies lacked previously mentioned aspects [34]. Only studies of high and good quality were considered for the review.

Most studies had clear research aims, the methodology and design were appropriate, and so were the data collection methods used. A detailed description of the recruitment process data analysis, findings, value of the research and contribution to practice was provided. Eleven (11) studies were appraised with the CASP tool, and two of the studies, Ackers et al. [35] and Burrowes et al. [18], did not meet the critical appraisal cut-off of 7/10 and were therefore excluded [36]. All four studies appraised with the JHNEBP tool were of good quality and thus included.

### Data extraction

The 13 relevant studies of good quality underwent data extraction by the researcher and an independent co-reviewer for correctness and relevance. The researcher developed a data extraction table to ensure the inclusion of all relevant findings that answered the review question [25, 36]. The headings are author/location, study focus, study findings, and findings relevant to this study.

### Data synthesis

Findings from the 13 studies were combined in the review. To synthesise data, similar and recurring concepts among studies were identified. Once identified the concepts were grouped according to similarity in meaning. Subsequently, descriptive, and analytical themes were used to create themes and sub-themes. The meta-synthesis involved three stages: (1) reading the text repeatedly, (2) developing descriptive themes and (3) generating analytical themes. The stages were according to the thematic analysis by Thomas and Harden [27].

### Stages one and two: coding and developing descriptive themes

Firstly, the researchers read the text repetitively to identify similar concepts among the primary studies before coding according to similarities in meaning. The process was stopped when no new concepts emerged that addressed the review question.

### Stage three: generating analytical themes

Secondly, concepts were grouped according to similarities. Thirdly, the concepts led to the generation of tabulated first and second-order concepts. The first order represented the main themes, and the second order the sub-themes. Three main themes regarding factors that influence respectful and humanising perinatal care practices, according to healthcare professionals, emerged. Lastly, the second-order concepts were assigned to the relevant themes as sub-themes (Table 3).

**Table 3:** Themes and sub-themes generated from the perspectives of healthcare professionals

Themes	Sub-themes
Institution-related factors	• Human resources • Medical resources • Norms and practices • Physical infrastructure
Healthcare provider-related factors	• Personal attributes • Interpersonal relationships • Competence
Women-related factors	• Knowledge • Preferences

### Characteristics of the included studies

The 13 studies met the inclusion criteria and were published in English. Three hundred ninety-three (393) HCPs working in diverse maternity settings participated in the studies. Five studies had samples of midwives only [37–41]  while the rest had midwives and doctors [42–49].

Eleven of the included studies were conducted on the African continent [37, 38, 41–43, 45–49], while for the other two studies one was conducted in Middle East Asia [40] and the other Southeast Asia [44]. No study from a high-income country was identified. Studies’ settings were both rural and urban. The healthcare facilities ranged from public/government hospitals, tertiary and non-teaching public hospitals, private and faith (mission) hospitals and health centres.

## Results

The study focused on midwives and doctors working in maternity settings. In this review, we used the term healthcare professional (HCP) unless a reference was made explicitly to either a midwife or a doctor.

The results are presented under three themes generated from the perspectives of healthcare professionals: healthcare institution, healthcare professionals and women, using sub-themes as the basis for the presentation. In some instances, quotations are cited to support findings.

### Factors related to the healthcare institution

Health institution-related factors are presented under the following four sub-themes: human resources, equipment and medical supplies, physical infrastructure, and norms and practices.

### Human resources

Human resource shortage was a significant concern, as reported by HCPs. The shortage was reported in ten studies [38, 39, 42–49]. The findings revealed the prevalence of human resource shortage in many settings. HCPs provide perinatal care to women; however, human resource shortage can compromise care if HCPs work extra hours to cover the shortage as this will lead to exhaustion [43]. Due to the high patient-midwife ratio, continuous support was not possible as midwives attended to more than one woman at a time [43, 44, 46].

### Medical resources

Medical resources refer to equipment and medical supplies that are needed to provide care efficiently and effectively. Narratives of inadequate equipment and beds prevailed [43, 48], and there was a lack of medical supplies, such as essential medicines and personal protective equipment, as reported in most of the included studies (38, 39, 40, 42–44, 48, 49]. Due to limited medical supplies [44, 47, 49] and sterile equipment, the process of sterilisation was sometimes shortened to hasten its availability for procedures [40]. Shortage of medical resources was prevalent in most settings - an aspect that can compromise patient safety.

### Norms and practices

Norms and practices influence the type of care HCPs render to women. The environment was highly medicalised, even for low-risk labouring women, and routine practices other than EBPs contributed to care that did not promote RPC among HCPs [38, 40].

*“Disrespect is a consequence of working in a medicalised context. They (obstetricians) treat labouring women hastily and completely medically. If we had midwife-led birth centres, then we could provide respectful care for women.”* [40].

HCPs in some of the studies [41, 43, 47] reported a lack of support from their superiors. Another revelation was that managers sometimes blamed HCPs for adverse outcomes [48]. Furthermore, lack of promotion and incentives for extra work and long working hours contributed to D&A [41, 43, 47].

*“Sometimes, you keep on doing and discharging your responsibilities appropriately, but no one from the senior managers comes to you and sees what you do and gives you recognition.”* [43].

Highlighted by HCPs was the lack of managers’ instituting redress on disrespectful practices [37, 41]. The lack thereof perpetuated uncaring practices.

Another factor raised by HCPs was that training only focused on women’s needs and not on HCPs’ welfare [41, 43]. HCPs felt that their needs were neglected as the management did not address the challenges they were experiencing.

### Physical infrastructure

The physical infrastructure must be adequate and well-maintained to ensure safety and security. Inadequate birthing-room space was prevalent, as was reported in nine studies [39, 40, 42, 44–49]. Allowing many people in a small area poses a health risk [48, 49]. To ensure privacy, birth companions are often requested to leave the ward before the examination of women [40, 43, 45]. Sometimes, only female birth companions are allowed during early labour but not during actual childbirth to maintain privacy [44]. Birth companionship is an essential component of RPC as it allows women to have the continuous present of a support person. There is also a shortage of suitable waiting areas [40].

*“The companions stay outside in a cold place, from night until morning. They lose their patience, so they may get easily nervous. If they could stay in a suitable place, they would be comfortable and cooperate with us to support the women.”* [40].

Limited physical space also prevented free mobility, a non-pharmacological pain relief method for labouring women [46].

Other challenges related to physical infrastructure are non-functioning ablution facilities, water shortage, poor ventilation [43] and dilapidated buildings that pose a health risk [47]. A woman can become anxious, stressed, and disrespected in an environment without water and good ventilation [40, 43, 46]. HCPs acknowledged the importance of a physically pleasant environment as it might make a woman relax.

### Factors related to healthcare professionals

Healthcare professional-related factors were discussed under the following: personal attributes and competence.

### Personal attributes

According to the HCPs, a pleasant character by the HCP was important in relaxing and boosting women’s confidence [40]. A display of kindness and compassion makes women feel acknowledged [37]. RPC entails making a woman feel at home in a healthcare facility and appreciate the care afterward [40]. Midwives value effective communication to facilitate good interpersonal relations with women [40, 41, 44]. Respectful care is about giving the right care to the woman; this can be achieved through effective communication [40]. Sharing information calmly would relax the woman [37, 40, 41]. Additionally, HCPs acknowledged that women should be treated equally, so that each woman can feel appreciated [38, 39, 41, 42, 49]. Communication and a pleasant character were acknowledged by some of the HCPs as aspects appreciated by women.

HCPs reported that harsh treatment and force were used and justified as a way of getting women to cooperate [41, 49]. According to midwives, they, too, sometimes get overwhelmed and focus on birth outcomes and not the mother’s emotional well-being hence the harsh treatment of women [40, 45, 48]. Another finding was the acknowledgment by HCPs that women should be treated fairly to promote good interpersonal relationships [37, 40, 41]. HCPs agreed that kindness [37] and a pleasant attitude [40] enabled women to relax.

Although analgesia is a necessity during childbirth, sometimes midwives withhold it as they rationalised pain as part of childbirth (41, 44, 46,47 48]. “Painful deliveries are the norm, and scripture and religious messaging on the pain inherent in childbirth” [41]. The lack of analgesia makes women restless and less cooperative during childbirth.

### Competence

HCPs acknowledged competence as key in providing RPC. Midwives acknowledged that RPC is “non-abusive, women-centred and respects childbearing women’s rights” [37]. HCPs are highly trained individuals [41, 45, 46] however the implementation of EBP was inconsistent. Intervention packages on RPC for HCP training might promote the use of EBPs such as non-discriminatory care, informed consent, mobilisation, and companionship during childbirth enabling positive experiences for women [40]. Midwives acknowledged that not all women need medical interventions and that scientific evidence should inform decisions [40]. However, in one study [38] it was reported that occasionally HCPs urged women to deliver in the lithotomy position, exposing them to discomfort and predisposing them to supine hypotension syndrome [38]. According to HCPs training on “rights of women’ contributed to improved relationships with women [42, 47].

*“I used to physically abuse women. But after I went through training on the rights of women, I had to change my attitude.”* [42].

Besides knowledge and skills, cultural competence is a crucial element in RPC. HCPs should be culturally oriented to render care congruent to women from diverse backgrounds [40, 45, 46]. Skilfulness by HCPs is necessary in providing safe and acceptable care for women.

### Factors related to women

Women-related factors influencing respectful perinatal care are discussed under the following sub-themes: knowledge and preferences.

### Knowledge

HCPs highlighted that the right to information is fundamental for women to make informed decisions [37, 42, 44, 45]. Timely preparation can be achieved through antenatal classes [38, 40, 43]. Antenatal preparation helps the mother to plan for the birth, identify her birth companion of choice and cope well during childbirth [40].

*“A provider on duty who was not directly involved in direct patient care was utilized to help translate.”* [45].

Information related to childbirth preparation is essential for women as childbirth can be stressful, especially if unprepared.

#### Preferences

Women have varying preferences, these are influenced by culture, traditional practices, and religious beliefs [39, 40, 46, 47]. While some opt for a birth companion, others might not. Also reported is the preferred gender for a HCP; in some cultures, men are not allowed to ‘deliver’ women [39]. Other requests, such as taking the placenta home, can easily be accommodated [46].

*“When they come here, the rights of women from every culture and tradition should be protected, and we must pay attention to them.”* [40].

HCPs agreed that women have varying preferences influenced by aspects such as culture, traditional practices, and religious beliefs.

## Discussion of findings

The current review revealed the various factors that contribute to respectful perinatal care. It is evident that administrative and healthcare professional factors play a major role in the type of perinatal care women receive. Shortage of healthcare professionals was predominant and was cited by HCPs as contributing to increased workload, burnout, stress, and demotivation [38, 39, 44, 46–49]. Due to the demotivation and exhaustion of HCPs, women might not receive optimal and individualised care. Thus, healthcare institutions need to address the gap in staffing norms and work distribution as this might boost HCPs’ morale. Still, under human resources, another revelation was the lack of promotion and incentives for extra work and long working hours that contributed to compromised care [41, 43, 47]. Therefore, performance awards as recognition for exemplary performance might contribute to the motivation of HCPs. Equally, the lack of consequences for disrespect contributed to non-adherence to respectful practices [37, 41]. Corrective measures need to be established for the mistreatment of women so that HCPs are held accountable for their actions.

Parallel to human resource shortage was inadequate equipment and medical supplies that contributed to sub-optimal patient care as reported in most of the studies [39, 38, 40, 42–44, 48, 49]. Some of the women slept on the floor, while others sat on chairs for a long time waiting for a bed to be available [38, 48]. It is therefore important to ensure that the equipment is adequate and well maintained. Conclusively, efficient, and effective perinatal care requires adequate equipment and medical supplies so that women can be afforded the care and comfort they rightfully deserve.

Another revelation for this review was that inadequate space and poorly maintained physical infrastructure compromised the provision of RPC as it posed a health and safety risk [39, 40, 44–49]. The inadequate space also resulted in overcrowding, limited mobility for women in early labour, and no room for birth companions [39, 40, 48, 49]. Mobility during childbirth has benefits such as a shorter duration of labour and pain relief [50]. Thus, mobilization might be beneficial for low-risk women if the space allows. WHO [13] also recommends adequate infrastructure to accommodate women and their birth companions for emotional and physical support. Thus, healthcare institution administrators should ensure that the infrastructure is adequate and well-maintained. More so, using affordable items such as screens to partition the room in resource-restrained settings might be useful in providing privacy for women.

Two studies [38, 40] in this review highlighted the need for HCPs to restrict the medicalization of childbirth and interventions in low-risk women. Supporting the review findings a study by Hastings-Tolsma [51] states that all HCPs within maternity settings must embrace EBPs such as non-medicalization of childbirth, use of interventions only when medically indicated and allowing childbirth companionship according to an individual woman’s needs.

Furthermore, this review highlighted that HCPs’ attitudes and behaviours contribute to the type of care rendered and, subsequently, women’s experiences [37, 40]. Empathy and sensitivity towards women might allay anxiety. Incidences of discrimination were reported in most studies [38, 39, 41, 42, 49]. Thus, positive partnerships between women and HCPs are vital in creating a conducive environment where both can co-exist amicably. The White Ribbon Alliance [52] emphasises that perinatal care should be non-discriminatory. Supporting women emotionally, physically, and informationally is essential for their confidence and well-being.

The importance of reliable information as a contributor to decision-making by women was also prevalent in this review [41–44, 47]. In the absence of relevant information, women cannot make autonomous decisions. HCPs cited illiteracy and language differences as barriers to the comprehension of information [41, 44]. Childbirth preparation and informed decisions are vital; however, language and illiteracy communication barriers might inhibit women from meaningful engagements and making informed decisions. Authors Akhavan and Edge [53] recommend using simpler terms or a familiar language as it would be appreciated by women. Thus, women should receive health information using simple terms in a language they understand [45].

Bohren et al. [54] emphasised the need for a thorough explanation for women to make informed decisions. Communication between HCPs and women is fundamental in perinatal settings to enable autonomy for women. According to this review’s findings HCPs can provide congruent care by possessing the necessary knowledge, skills, and attitudes aligned with EBPs [46]. Thus, ongoing in-service training on EBP in the context of RPC might result in optimal health outcomes for mothers and babies as well as positive experiences for women.

The World Health Organization [13] recommends skilled birth attendants to safeguard the welfare of women during childbirth. In the review, it was revealed that training on RPC helped HCPs improve their competence towards providing dignified care [37, 43, 46, 47]. Thus, training on value clarification can be successful by making HCPs more aware of RPC. The review reiterates that women are diverse individuals, and so are their wants and needs an aspect HCPs should pay attention to when providing care [37, 40, 45]. Furthermore, culture, religion, and education influence women’s choices [39, 46]. More so, women should be acknowledged as individuals with varying needs [37, 40, 45]. However, accommodating women’s wishes depends on the implication for maternal and fetal well-being [45]. Thus, HCPs should maintain a balance between a woman’s preferences and the possible consequences concerning EBP.

### Limitation

While this study was able to explore and describe factors that affect RPC according to HCPs, some studies might have been missed due to incorrect indexing.

## Conclusion

The purpose of the study was to gather, appraise, and synthesise evidence on the factors influencing respectful perinatal care provided by healthcare professionals. This was achieved by exploring and synthesising findings from various studies that examined the perceptions of doctors and midwives regarding the factors affecting the provision of respectful perinatal care. In this review, three themes were discussed under the respective sub-themes: institutional-related, healthcare-related, and women-related factors. Factors that facilitate respectful perinatal care in this review were identified as adequate human and physical resources and medical supplies, healthcare provider knowledge, skills, and attributes [38–49].

The thirteen studies included in the systematic review highlighted the importance of addressing perinatal care related issues within the context of the practice environment.

### Electronic supplementary material

Below is the link to the electronic supplementary material.

Supplementary Material 1

## Data Availability

All data analysed during this study are included in this published article [and its supplementary information files].

## Abbreviations

AND: Academy for Nutrition and Dietetics
CRD: Centre for Reviews and Dissemination
D&A: Disrespect and Abuse
EBP: Evidence-Based Practice
EPPI: Evidence for Policy and Practice Information
HCP: Healthcare Professional
HRW: Human Rights Watch
JHNEBP: Johns Hopkins Nursing Evidence-Based Practice
RPC: Respectful Perinatal Care
WHO: World Health Organization
SPIDER: Sample, Phenomenon of Interest, Design, Evaluation, Research type
PRISMA: Preferred Reporting Items for Systematic Reviews and meta Analyses
RIS: Research Information Systems
CASP: Critical Appraisal Skills Programme
WRA: White Ribbon Alliance
