# Supplementary material for: Factors influencing respectful perinatal care among healthcare professionals in low-and middle-resource countries: a systematic review
Source: BMC Pregnancy Childbirth. 2024 Jun 24;24:442. doi: 10.1186/s12884-024-06625-6 (PMC11194958; doi:10.1186/s12884-024-06625-6)
Supplement: Supplementary file 1 — Supplementary Material 1 [file 12884_2024_6625_MOESM1_ESM.docx]

**SUPPLEMENTARY FILE**

**Additional file 1: PRISMA flow chart of the systematic review**

| References identified through database search =1761  References identified through manual search.  =21  **Identification**  Duplicates  N =111  N=1782  **Screening**  Not relevant  N=1649  N=1670  Full-text articles excluded according to exclusion criteria.  N=6  Full text records screened for eligibility.  N=21  (n=)  **Eligibility**  E  2 studies excluded on methodological quality and ethical considerations.  Studies included in critical appraisal=N15  **Included**  Studies included in the synthesis.  N= 13 |
| --- |

**Additional file 2: CASP tool: Critical appraisal of legible studies**

|  | **Authors/title/ bibliographic information** | **Type of study design, setting and language of data collection** | **Sampling, sample size and demographics** | **Data collection technique and analysis** | **Rigour** |
| --- | --- | --- | --- | --- | --- |
| 1. | Ackers, L., Webster, H., Mugahi, R. & Namiiro, R. 2018. "What price a welcome? Understanding structure agency in the delivery of respectful midwifery care in Uganda", *International Journal of Health Governance*, 23:(1)46-59. <https://doi.org/10.1108/IJHG-11-2017-0061> | Design: Qualitative but not further specified  Setting: Public health facilities, Uganda  Language: English | Sampling: Purposive  Sample size: Not provided.  Healthcare professionals:   - Midwives   Age: Not provided  Experience: Not provided | Data collection: In-depth interviews and focus groups.  Data analysis: content | 1. Statement of aims of research: clear. 2. Methodology: **not discussed** 3. Research design: Appropriate to address aims. 4. Recruitment: **not clear** 5. Data collection method: appropriate. 6. Relationship between researcher and participants (reflexivity): **not discussed.** 7. Ethical issues: **insufficient** 8. Data analysis: **insufficient** 9. Findings: a clear statement provided. 10. Value of research: discussed and its contribution to practice and the need for further research indicated.   **C/A = 5/10**  **Excluded** |
| 2. | Burrowes, S., Holcombe, S.J., Jara, D., Carter, D. & Smith, K. 2017. Midwives' and Patients' Perspectives on Disrespect and Abuse During Labor and Delivery Care in Ethiopia: A Qualitative Study. *BMC Pregnancy and Childbirth,* 17:263. [DOI 10.1186/s12884-017-1442-1](file:///C:\Users\10387323\Dropbox\My%20PC%20(p10387323-1)\Documents\GroupWise\DOI%2010.1186\s12884-017-1442-1) | Design: Qualitative - exploratory  Setting: Debre Markos, a city in  Amhara region located 5 hours northwest of Ethiopia’s capital.  Language: Amharic and English | Sampling: Purposive  Sample size: 4  Healthcare professionals:   - 4 Midwives   Age: 18-30  Experience: Minimum 2 years | Data collection: In-depth-interviews  Data analysis: Thematic | 1. Statement of aims of research: clear. 2. Methodology: **insufficient midwives** 3. Research design: appropriate to address aims. 4. Recruitment: **not detailed** 5. Data collection method: **insufficient** 6. Relationship between researcher and participants (reflexivity): **not discussed.** 7. Ethical issues: **insufficient** 8. Data analysis: sufficiently discussed. 9. Findings: a clear statement provided. 10. Value of research: discussed and its contribution to practice and the need for further research indicated.   **C/A =5/10**  **Excluded** |
| 3. | Dzomeku, V.M., Mensah, B.A.B., Nakua, E.K., Agbadi, P., Lori, J.R. & Donkor, P. 2020a. Exploring midwives' understanding of respectful maternal care in Kumasi, Ghana: Qualitative inquiry. *PLoS*  *ONE,* 15(7): e0220538. <https://doi.org/10.1371/>  [journal.pone.0220538](https://doi.org/10.1371/) | Design: Qualitative exploratory descriptive  Setting: A tertiary health facility in Kumasi, located in the Ashanti Region, the central part of Ghana.  Language: English | Sampling: Purposive  Sample size: 15  Healthcare professionals:   - Midwives   Age: 31-48 years  Experience: Average 8 years | Data collection: Semi-structured interviews.  Data analysis: Thematic | 1. Statement of aims of research: clear. 2. Methodology: appropriate and sufficiently discussed. 3. Research design: appropriate to address aims. 4. Recruitment: discussed 5. Data collection method: appropriate. 6. Relationship between researcher and participants (reflexivity): **not discussed.** 7. Ethical issues: discussed. 8. Data analysis: sufficiently discussed. 9. Findings: a clear statement provided. 10. Value of research: discussed and its contribution to practice and the need for further research indicated.   **C/A = 9/10**  **Included** |
| 4. | Dzomeku, V.M., Mensah, A.B.B., Nakua, E.K., Pascal Agbadi, P., Lori, J.R. & Donkor, P. 2020b. "I wouldn't have hit you, but you would have killed your baby:" exploring midwives' perspectives on disrespect and abusive Care in Ghana. *BMC Pregnancy and Childbirth,* 20:15. https://doi.org /10.1186/s12884-019-2691-y | Design: Qualitative exploratory descriptive  Setting: a tertiary health facility in Kumasi, located in the Ashanti region of Ghana  Language: English | Sampling: Purposive  Sample size: 15  Healthcare professionals: Midwives  Age: 31-48  Experience: Average 8 years | Data collection: In-depth interviews using a semi-structured interview guide.  Data analysis: Thematic | 1. Statement of aims of research: clear. 2. Methodology: appropriate and sufficiently discussed. 3. Research design: appropriate to address aims. 4. Recruitment: **Detailed** 5. Data collection method: appropriate. 6. Relationship between researcher and participants (reflexivity): **Not discussed.** 7. Ethical issues: Discussed 8. Data analysis: sufficiently discussed. 9. Findings: a clear statement provided. 10. Value of research: discussed and its contribution to practice and the need for further research indicated.   **C/A = 9/10**  **Included** |
| 5. | Lusambili, A., Wisofschi, S., Shumba,C., Obure, J., Mulama, K., Nyaga , L., … Temmerman, M. 2020. Health care workers' perspectives of the influences of disrespectful maternity care in rural Kenya. *International Journal of Environmental* *Research and Public Health,* 17: 8218. <doi:10.3390/ijerph17218218> | Design: Qualitative descriptive  Setting: Kilifi and Kisii are two of the poorest counties in Kenya  Language: English | Sampling: Purposive  Sample size: 24  Healthcare professionals: Nurses  Age: Not specified  Experience: At least one year | Data collection: Semi-structured interview guide.  Data analysis: Thematic | 1. Statement of aims of research: clear. 2. Methodology: appropriate and sufficiently discussed. 3. Research design: appropriate to address aims. 4. Recruitment: Not d**etailed** 5. Data collection method: appropriate. 6. Relationship between researcher and participants (reflexivity): **Not discussed.** 7. Ethical issues: Discussed 8. Data analysis: sufficiently discussed. 9. Findings: a clear statement provided. 10. Value of research: discussed and its contribution to practice and the need for further research indicated.   **C/A = 8/10**  **Included** |
| 6. | Maung, T.M., Lwin, K., Mon, N.O., Tunçalp, Ö,. Aye, S.N., Soe., Y.Y. & Bohren, M.A. 2020. A qualitative study on acceptability of the mistreatment of women during childbirth in Myanmar. *Reproductive Health*, 17:56. <https://doi.org/10.1186/s12978-020-0907-2> | Design: Qualitative descriptive  Setting: Bago and Taungoo Townships, Myanmar  Language: Myanmar language and English | Sampling: Purposive  Sample size: 25  Healthcare professionals:   - 16 nurses - 9 doctors   Age: 20 – 50 years plus  Experience: 0-15 years plus | Data collection: In-depth interviews using a semi-structured interview guide.  Data analysis: Thematic | 1. Statement of aims of research: clear. 2. Methodology: appropriate and sufficiently discussed. 3. Research design: appropriate to address aims. 4. Recruitment: **Not** **detailed** 5. Data collection method: appropriate. 6. Relationship between researcher and participants (reflexivity): **Not discussed.** 7. Ethical issues: Minimal discussion 8. Data analysis: sufficiently discussed. 9. Findings: a clear statement provided. 10. Value of research: discussed and its contribution to practice and the need for further research indicated.   **C/A = 7/10**  **Included** |
| 7. | Moridi, M., Pazandeh, F., Hajian, S. & Potrata. 2020. Midwives' perspectives of respectful maternity care during childbirth: A qualitative study. *PLoS ONE*, 15(3): e0229941. <https://doi.org/>  [10.1371/journal.pone.0229941](https://doi.org/) | Design: Qualitative but not specified  Setting: Two non-teaching public hospitals in Tehran, Iran  Language: Persian and English | Sampling: Purposive  Sample:17  Healthcare professionals:   - 17 staff midwives   Age:24-58 years  Experience: 1 – 29 years | Data collection: Semi-structured in-depth interviews  Data analysis: Content | 1. Statement of aims of research: clear. 2. Methodology: **Insufficiently discussed.** 3. Research design: Appropriate to address aims. 4. Recruitment: Clear 5. Data collection method: appropriate. 6. Relationship between researcher and participants (reflexivity): **Not discussed.** 7. Ethical issues: Sufficiently discussed. 8. Data analysis: Sufficiently discussed. 9. Findings: a clear statement provided. 10. Value of research: discussed and its contribution to practice and the need for further research indicated.   **C/A = 8/10**  **Included** |
| 8. | Mselle, L.T., Kohi, T.W. & Dol, J. 2018. Barriers and facilitators to humanising birth care in Tanzania: findings from semi-structured interviews with midwives and obstetricians. *Reproductive Health,* 15:137.  https://doi.org/10.1186/s12978-018-0583-7 | Design: Qualitative - exploratory  Setting: 2 District hospitals in the Mwanza and Mara regions in the Lake Zone, Tanzania  Language: Kiswahili and English | Sampling: Purposive  Sample size: 8  Healthcare professionals:   - 6 Midwives - 2 Obstetricians   Age: Not provided  Experience: Minimum 2 years | Data collection: Semi-structured interviews and FGDs  Data analysis: Thematic | 1. Statement of aims of research: clear. 2. Methodology: appropriate and sufficiently discussed. 3. Research design: appropriate to address aims. 4. Recruitment: **Not detailed** 5. Data collection method: appropriate. 6. Relationship between researcher and participants (reflexivity): **Not discussed.** 7. Ethical issues: **Minimal discussion** 8. Data analysis: sufficiently discussed. 9. Findings: a clear statement provided. 10. Value of research: discussed and its contribution to practice and the need for further research indicated.   **C/A =7/10**  **Included** |
| 9. | Oluoch-Aridi, J., Smith-Oka, V., Milan, E. & Dowd, D. 2018.  Exploring mistreatment of women during childbirth in a peri-urban setting in Kenya: experiences and perceptions of women and healthcare providers. *Reproductive Health,* 15:209  https://doi.org/10.1186/s12978-018-0643-z | Design: Qualitative descriptive  Setting: Public maternity hospitals, health centres, private and faith-based health facilities in Kenya  Language: Swahili and English | Sampling: Purposive  Sample: 14  Healthcare professionals:   - 6 doctors - 8 midwives   Age: Not provided  Experience: Not provided | Data collection: Semi-structured in-depth interview guide  Data analysis: Thematic | 1. Statement of aims of research is clear. 2. Methodology: appropriate. 3. Research design: is appropriate to address aims. 4. Recruitment: process discussed. 5. Data collection method: appropriate. 6. Relationship between researcher and participants (Reflexivity): **Cannot tell**. 7. Ethical issues: Discussed 8. Data analysis: Sufficiently discussed. 9. Findings: a clear statement provided. 10. Value of the research: Contribution and implications for practice are discussed.   **CA 9/10**  **Included** |
| 10 | Smith, J., Banay, R., Zimmerman, E., Caetano, V., Musheke, M. & Kamanga, A. 2020. Barriers to provision of respectful maternity care in Zambia: results from a qualitative study through the lens of behavioral science. *Pregnancy and Childbirth*, 20:26.  https://doi.org/10.1186/s12884-019-2579-x | Design: Qualitative not specified  Setting: Chipata district, Eastern  Province, Zambia.  Language: English | Sampling: Purposive  Sample size: 17  Healthcare professionals: Nurses  Age: Not provided  Experience: Not provided | Data collection: In-depth interviews  Data analysis: Thematic | 1. Statement of aims of research: clear. 2. Methodology: appropriate and sufficiently discussed. 3. Research design: Appropriate to address aims. 4. Recruitment: **not detailed** 5. Data collection method: appropriate. 6. Relationship between researcher and participants (reflexivity): **not discussed.** 7. Ethical issues: **considered mostly.** 8. Data analysis: sufficiently discussed. 9. Findings: a clear statement is provided. 10. Value of research: discussed and its contribution to practice and the need for further research indicated.   **C/A = 7/10**  **Included** |
| 11. | Warren, C.E., Njue, R., Ndwiga. & Abuya, T. 2017. Manifestations and drivers of mistreatment of women during childbirth in Kenya: implications for measurement and developing interventions. *BMC Pregnancy and Childbirth*, 17:102. DOI 10.1186/s12884-017-1288-6 | Design: Qualitative descriptive  Setting: Four facilities rural and nine urban or peri-urban areas, in Kenya  Language: Swahili and English | Sampling: Purposive  Sample size: 11  Healthcare professionals:   - 11 doctors and midwives   Age: Not provided  Experience: Not provided | Data collection: In-depth interviews using a semi-structured interview guide.  Data analysis: Thematic | 1. Statement of aims of research: clear. 2. Methodology: appropriate and sufficiently discussed. 3. Research design: appropriate to address aims. 4. Recruitment: **not** d**etailed** 5. Data collection method: appropriate. 6. Relationship between researcher and participants (reflexivity): **not discussed.** 7. Ethical issues: discussed. 8. Data analysis: sufficiently discussed. 9. Findings: a clear statement provided 10. Value of research: discussed and its contribution to practice and the need for further research indicated.   **C/A = 8/10**  **Included** |

**Additional file 3: JHNEBP tool: Critical appraisal for mixed-methods studies**

|  | **Authors/title/ bibliographic information** | **Type of study design, setting and language of data collection** | **Sampling, sample size and demographics** | **Data collection technique and analysis** | **Rigour** |
| --- | --- | --- | --- | --- | --- |
| 1 | Afulani, P.A., Kelly, A.M., Buback, L., Asunka, J., Kirumbi, L. & Lyndon, K. 2020. Providers' perceptions of disrespect and abuse during childbirth: a mixed-methods study in Kenya. Health Policy and Planning, 35:577–586. doi: 10.1093/heapol/czaa009 | Design: Convergent mixed methods  Setting: a rural county in Western Kenya Language: English, Swahili, and Luo | Sampling: Purposive  Sample size: 32  Healthcare professionals:   - 25 Nurse/midwife - 7 Clinical officer/doctor   Age: Below 30 – above 39 years  Experience: More than 10 years | Data collection: structured and open-ended questions and interviews  Data analysis: Thematic | 1. Does the researcher identify what is known and not known about the problem and how the study will address any gaps in knowledge? Yes 2. Was the purpose of the study clearly presented? Yes 3. Was the literature review current (most sources within last 5 years or classic)? Yes 4. Was the sample size sufficient based on the study design and rationale? Yes 5. If there is a control group: N/A  - Were the characteristics and/or demographics similar in both the control and intervention groups? - If multiple settings were used, were the settings similar? - Were all groups equally treated except for the intervention group(s)  1. Are data collection methods described clearly? Yes 2. Were the instruments reliable (Cronbach’s α [alpha] > 0.70)? N/A 3. Was instrument validity discussed? N/A 4. If surveys/questionnaires were used, was the response rate > 25%? N/A 5. Were the results presented clearly? Yes 6. If tables were presented, was the narrative consistent with the table content? Yes 7. Were study limitations identified and addressed? Yes 8. Were conclusions based on results? Yes   **C/A: Good quality**  **Included** |
| 2. | Asefa, A., McPake., B., Langer, A., Bohren, M.A. Morgan, A. 2020. Imagining maternity care as a complex adaptive system: understanding health system constraints to the promotion of respectful maternity care. *Sexual and Reproductive Health Matters,* 28:1, e1854153.  DOI:10.1080/26410397.2020.1854153 | Design: Mixed methods intervention  Setting: Three public hospitals  (two general and one primary hospital), Ethiopia  Language: Amharic and English | Sampling: Purposive  Sample size: 53  Healthcare professionals:   - 47 midwives - 3 nurses - 1 general practitioners - 2 health officers   Age: Not provided  Experience: Not provided | Data collection: Postal survey (semi-structured questionnaire)  Data analysis: Content and statistical | 1. Does the researcher identify what is known and not known about the problem and how the study will address any gaps in knowledge? Yes 2. Was the purpose of the study clearly presented? Yes 3. Was the literature review current (most sources within last 5 years or classic)? Yes 4. Was the sample size sufficient based on the study design and rationale? Yes 5. If there is a control group: N/A  - Were the characteristics and/or demographics similar in both the control and intervention groups? - If multiple settings were used, were the settings similar? - Were all groups equally treated except for the intervention group(s)  1. Are data collection methods described clearly? Yes 2. Were the instruments reliable (Cronbach’s α [alpha] > 0.70)? N/A 3. Was instrument validity discussed? N/A 4. If surveys/questionnaires were used, was the response rate > 25%? N/A 5. Were the results presented clearly? Yes 6. If tables were presented, was the narrative consistent with the table content? Yes 7. Were study limitations identified and addressed? Yes 8. Were conclusions based on results? Yes   **C/A: Good quality**  **Included** |
| 3. | Moyer, C.A., McNally, B., Aborigo, R.A., Williams, J.E.O. & Afulani, P. 2021. Providing respectful maternity care in northern Ghana: A mixed-methods study with maternity care providers. Midwifery 94:102904. <https://doi.org/10.1016/j.midw.2020.102904> | Design: Mixed methods cross-sectional  Setting: Government health facilities in rural northern Ghana  Language: English | Sampling: Purposive  Sample size: 31  Healthcare professionals:   - 28 midwives - 3 doctors   Age: Mean -38.6 years  Experience: ≤ 5 years | Data collection: Semi-structured interviews  Data analysis: Thematic and statistical | 1. Does the researcher identify what is known and not known about the problem and how the study will address any gaps in knowledge? Yes 2. Was the purpose of the study clearly presented? Yes 3. Was the literature review current (most sources within last 5 years or classic)? Yes 4. Was the sample size sufficient based on the study design and rationale? **No** 5. If there is a control group: N/A  - Were the characteristics and/or demographics similar in both the control and intervention groups? - If multiple settings were used, were the settings similar? - Were all groups equally treated except for the intervention group(s)  1. Are data collection methods described clearly? Yes 2. Were the instruments reliable (Cronbach’s α [alpha] > 0.70)? N/A 3. Was instrument validity discussed? N/A 4. If surveys/questionnaires were used, was the response rate > 25%? N/A 5. Were the results presented clearly? Yes 6. If tables were presented, was the narrative consistent with the table content? Yes 7. Were study limitations identified and addressed? Yes 8. Were conclusions based on results? Yes   **C/A: Good quality**  **Included** |
| 4. | Ndwiga, C., Warren, C.E., Ritter, J., Sripad, P. & Abuya, T. 2017. Exploring provider perspectives on respectful maternity care in Kenya: "Work with what you have." *Reproductive Health,* 14:99.  [DOI 10.1186/s12978-017-0364-8](file:///C:\Users\10387323\Dropbox\1%20Besig\Studente%20werk\Lunda,%20P\Ch%203%20send%2010%20March\DOI%2010.1186\s12978-017-0364-8) | Design: Mixed methods intervention  Setting: 13 health facilities in 5 counties (Private, public & faith-based) Kenya  Language: English | Sampling: Purposive  Sample:136   - Baseline n=67 - End line n=69   Healthcare professionals:   - Nurse-midwives =122 - Doctors =14   Age: Average 35 years  Experience: Average 11 years | Data collection: Semi-structured interviews  Data analysis: Content and statistical | 1. Does the researcher identify what is known and not known about the problem and how the study will address any gaps in knowledge? Yes 2. Was the purpose of the study clearly presented? Yes 3. Was the literature review current (most sources within last five years or classic)? Yes 4. Was the sample size sufficient based on the study design and rationale? **To some extent** 5. If there is a control group: N/A  - Were the characteristics and/or demographics similar in both the control and intervention groups? - If multiple settings were used, were the settings similar? - Were all groups equally treated except for the intervention group(s)  1. Are data collection methods described clearly? Yes 2. Were the instruments reliable (Cronbach’s α [alpha] > 0.70)? N/A 3. Was instrument validity discussed? N/A 4. If surveys/questionnaires were used, was the response rate > 25%? N/A 5. Were the results presented clearly? Yes 6. If tables were presented, was the narrative consistent with the table content? Yes 7. Were study limitations identified and addressed? Yes 8. Were conclusions based on results? Yes   **C/A: Good quality**  **Included** |

**Additional file 4: Populated data extraction table of included studies**

| **Author/location** | **Study focus** | **Study's findings** | **Findings relevant to this study** |
| --- | --- | --- | --- |
| Perspectives of healthcare professionals as relevant to the review question led to developing themes and sub-themes about factors influencing maternity care practice. The factors are either institutional, healthcare professional-related related or woman-related. | | | |
| Afulani *et al.* 2020  Kenya | Examined the extent and drivers of disrespect and abuse during facility-based childbirth from the perspectives of maternity care providers in a rural county in Kenya. | The study was part of a “larger mixed-methods project in a rural county in western Kenya to understand community perceptions of quality of maternity care.” The study’s findings were discussed under:  Drivers of disrespect and abuse with five themes: 1) difficult and uncooperative women, 2) environmental and situational factors, 3) provider attitudes, 4) provider bias and provider, 5) training and women’s empowerment.   1. **Difficult and uncooperative women**   Healthcare professionals justified verbal and physical abuse to get women to cooperate for fear of complications. The justification was that the use of ‘force’ was the last option for the woman’s own good and the baby. **Environmental and situational factors**   - **Stressful work conditions and burnout**   HCP cited exhaustion, high work overload, and stress as contributory impatience, bad temperament, and frustration. Other reasons for the frustration were attacks by women’s relatives, a language barrier, and unsupportive colleagues.   - **Facility culture and accountability**   The absence of repercussions for disrespectful behaviour contributed to the continued mistreatment of women.   - **Poor infrastructure and lack of supplies and medications**   Limited physical space compromised the privacy and confidentiality of women. Furthermore, the unavailability of screens worsened the problem. Medication and sanitary pads were also not available.   1. **Provider attitudes**   Poor HCP behaviours were attributed to work-related stress due to high workload.   - **Provider bias and provider**   Social standing influenced the care a woman received, close relations and literacy. Women from the upper class were treated more respectfully than those from the lower classes.   - **Training and women’s empowerment**   Skills training on respectful care, handling difficult women and human rights was necessary.  The authors concluded that respectful care depends on multifactorial issues ranging from the practice environment, societal expectations, healthcare professional behaviour and organizational culture (Afulani *et al.,* 2020:579-584) | **Institutional factors**  **Human resources**  Shortage of HCP resulted in an increased workload, physical and mental fatigue, and poor work output.  HCPs are blamed by superiors for adverse outcomes.  **Equipment and medical supplies**  Inadequate medical supplies, equipment and drugs compromised the quality of care. This frustrated HCPs, as they could not work effectively.  **Infrastructure**  Adequate physical space is required to ensure privacy, confidentiality and prevent overcrowding. |
|  |  |  | **Healthcare professional factors**  **Attributes**  Inability to cope with workload lead to bad temperament resulting in uncaring behaviours towards women.  HCPs know that compassionate and caring attributes represent RMC, but they were sometimes harsh towards women due to work-related factors. Discriminatory care for women does not represent RMC, as it violates women’s rights.  Lack of accountability and redress perpetuated in a culture of D&A. Due to the lack of consequences, HCPs normalised the mistreatment of women.  **Competence**  Training on RMC for midwives precedes competencies to provide RMC. |
| Asefa *et al.,* 2020  Ethiopia | Investigated the health system constraints to RMC in three Southern Ethiopian hospitals | This study is part of a “broader mixed-methods intervention study which was conducted to explore health system factors influencing RMC and to develop and assess mitigation approaches in southern Ethiopia.”  The following were revealed.   1. **Impact of RMC training on providers**   Participants reported having insight into RMC, such as obtaining informed consent, no physical or verbal abuse, neglect and abandonment of women. They became more aware of respectful care by reflecting on their behaviour before training and undignified practices.   1. **Providers perception of the training**   The participants narrated that they learned more about RMC. Some of the HCPs found the right to refusal of treatment by women unsettling as this did not result in patients receiving optimal care. Thus, women needed a thorough education.   - **Training methods**   The participants appreciated the methods used, i.e., "role-play, case scenarios, video shows and participatory." The different skill mixes and hierarchies of trainers were valued.   - **Concern for providers**   The concern was that the focus was more on upholding women's rights than care providers. Another aspect was the training manuals, guidelines and standard operating procedures that focused on the responsibilities of HCPs towards women but nothing on incentives and recognition of providers. Participants found it demotivating.   1. **Challenges in implementing RMC guidelines.**  - **Inadequate physical space and medical resources**   Supplies such as screens were not enough to provide privacy to women. Permitting birth companionship after the training on RMC leads to overcrowding.  Other medical supplies and equipment were inadequate to meet the units' needs. In certain hospitals, there was no water for the ablution block, which posed a health and safety hazard.   - **High workload**   Limited midwives lead to being overworked; an aspect midwives complained about. The absence of support persons meant that midwives had to provide constant care to women.  **Women's ignorance of appropriate care**  Due to a lack of knowledge, some women refuse to be examined or have an episiotomy performed, which requires education during the antenatal period.   - **Improving infrastructure and medical supplies**   The HCP requested constant availability of services, resources and adequate supplies and refurbishing dilapidated physical structures.   - **Empowerment on RMC and motivation**   The HCP suggested that training on RMC needed extension to support staff nursing students and medical interns for consistent, respectful practices.  HCP raised the need for more staff and improved the salary scale.   - **Engaging key stakeholders**   To ensure compliance with RMC, HCP indicated that including administrators, managers, support staff, academic institutions, tertiary hospitals, women, and communities was necessary.  The authors concluded that, though HCPs received training positively, there was still a need to strengthen the healthcare system (Asefa *et al.,* 2020:6-11). | **Institutional factors**  **Human resources**  A shortage of midwives leads to increased workload, long working hours and poor work output, compromising patient care.  Frustration due to a lack of human resources.  Participants also demanded employing more staff and improving the salary scale.  Lacking recognition for extra workload was demotivating incentives and recognition of providers and compromised patient care.  **Equipment medical supplies**  Inadequate supplies such as screens were not enough to provide privacy to women, so women were exposed, thereby compromising respectful care.  The health professionals demanded constant availability of services, resources, and adequate supplies to ensure quality care.  **Infrastructure**  The health professionals demanded refurbishing dilapidated physical structures to ensure adequate space to provide privacy. |
|  |  |  | **Healthcare professional factors**  **Attributes**  Healthcare providers were sensitive to women’s needs and thus treated them with care, respect, and dignity to avoid misunderstanding.  Effective communication with women and communities is vital to ensure sustained relationships. High workload contributed to strained interpersonal relationships with women.  **Competence**  Knowledge and skills enable the provision of RMC. Training on RMC provided participants with more insight. They became more aware, reflected on their own behaviour before training, and corrected undignified practices. "Role-play, case scenarios, video shows and participatory."  Birth companionship was acknowledged post-RMC training. |
|  |  |  | **Factors related to women**  **Knowledge**  Women need health information during the antenatal period about pregnancy and childbirth to make informed decisions.  Similarly, provision of role clarification of the support person during the antenatal period to avoid role confusion between the support person and midwife.  Families and communities need to be on board through health education. |
| Dzomeku *et al.* 2020a.  Ghana | Explored midwives' awareness of respectful maternity care. | The study findings were discussed under three themes**.**   1. **Awareness of RMC**   These were some of the midwives' perceptions of RMC "respectful care, non-abusive care, ensuring childbearing women-centred care, and respecting childbearing women's rights."   - **Respectful care**   Included informed consent, information provision and physical support to a labouring woman.  Non-abusive care  It meant ensuring women's emotional and psychological well-being and the absence of verbal or physical abuse.   - **Client-centred care**   Treating each woman according to individual needs  Respecting Childbearing women's rights  Inclusive of informed consent, privacy, and confidentiality, having a companion of choice and health information.   1. **Motivations for RMC**   The reasons for motivation to provide RMC included.  Perceived connection between the mother's treatment and neonatal outcomes  It was essential to ensure optimal well-being for the mother.   - **God's calling**   Midwives perceived being a midwife as a calling and not just a job.   - **Economic, social, and psychological benefits**   Acknowledgement and appreciation by the women they cared for were fulfilling and rewarding.   - **Midwives' experience of own labour**   Own childbirth experience made the midwife sensitive towards the women.   1. **Labour and pain management education**   Midwives narrated the need for women's education on pain management starting during the antenatal period so that women are adequately prepared psychologically.  Participants showed a disconnect between awareness and practice of RMC. Hitting women's thighs during 'bearing down was not seen as a ‘wrong or right’ practice. They could not justify it with EBP either.  The authors concluded that though midwives were somewhat aware of RMC, they still justified hitting women's thighs during childbirth. The authors recommended regular in-service training for the midwives, supervision, and an award system for recognition as motivation (Dzomeku *et al.,* 2020a: 12-14). | **Institutional factors**  **Human resource**  Lack of redress for non-adherence to RMC perpetuated non-compliance |
|  |  |  | **Healthcare professional**  **Attributes**  Midwifery was a “calling,” not just any other job. Empathy and sensitivity are a need.  Healthcare professionals acknowledged the importance of upholding women’s rights, women-centred care, and non-abusive care as a representation of RMC.  Good interpersonal relations are a necessity in ensuring respectful care.  Interpersonal relations were not always good; midwives justified hitting women's thighs during delivery to get the woman to cooperate.  Midwives could not give scientific evidence to support hitting women's thighs during "bearing down." This practice does not constitute RMC.  **Competence**  Midwives were aware of the components of RMC: informed consent, privacy, and confidentiality, having a companion of choice and health information, and client-centred, non-abusive care as a positive indicator of the provision of RMC.  Midwives were motivated to provide RMC to ensure positive outcomes.  Midwives acknowledged that competence is vital in ensuring optimal well-being for mother and baby. Besides competency, commitment also contributes to the provision of optimal care. |
|  |  |  | **Women related factors**  **Knowledge**  Lack of knowledge by women of childbirth compromises women’s ability to exercise autonomy. HCPs should provide information during the antenatal period to prepare women for childbirth and encourage decision-making involvement. |
| Dzomeku *et al.* 2020b  Ghana | Explored the experiences and views of midwives on disrespectful and abusive maternal care in their professional practice. | The study revealed that though midwives were aware of the elements of RMC, they still justified the D&A of women.   1. **Substandard care**   Midwives acknowledged substandard care constituted disrespect and abuse. Substandard care was cited as inclusive of unconsented care, discriminatory care, and disrespecting childbearing women’s rights of confidentiality and anonymity.”   1. **Non-evidenced based practices**   Healthcare professionals justified the D&A by recounting that sometimes it is because women are uncooperative and do not follow instructions, so force had to be applied.   1. **Health system constraints**   Human resource shortage led to high stress levels, exhaustion, demotivation, and aggression towards women.  Besides human resources, the medical supplies and equipment were insufficient. Worse still, the physical infrastructure did not enable privacy and confidentiality.  The authors concluded midwives knew what constitutes D&A. Revealed contributed to D&A (Dzomeku *et* *al.,* 2020b:4-10). | **Institutional factors**  **Human resources**  Adequate healthcare personnel are essential in providing one-to-one care and continuous support to women. Due to the shortage, the healthcare professionals are exhausted, stressed and agitated, resulting in verbal abuse towards women.  **Equipment and medical supplies**  Inadequate resources such as medical supplies and equipment compromise dignity. In some instances, beds were not enough; thus, women ended up sitting on chairs while waiting for the next bed to be available. |
|  |  |  | **Healthcare professional factors**  **Attributes**  **An unpleasant attitude attributed to a** high workload may have led to aggression towards women. The abuse was sometimes justified to get women to cooperate and follow commands.  Although midwives were aware of women’s rights, they still mistreated and discriminated against women, violating them. There is a need for training for RMC. |
|  |  |  | **Women**  **Knowledge**  Women need information about childbirth and expectations from them to cooperate and practice autonomy. |
| Lusambili *et al*. 2020 Kenya | Examined health care workers' (HCWs) perspectives on factors influencing DMC experienced by pregnant women at health care facilities. | The study identified four major contributors to disrespectful maternity care.   1. **Physical infrastructure challenges**   The physical environment should be conducive to functional equipment and adequate space to ensure privacy for women. However, the midwives stated that the area was inadequate for the number of women using the services.   1. **Medical equipment**   Basic equipment is a necessity in the provision of RMC. Without adequate resources, nurses mentioned that compromised patient care, as they could not meet the needs of the women.   1. **Understaffing**   Shortage of midwives and high patient-midwife ratios were identified as barriers to providing RMC due to increased workload, resulting in stress, burnout, and exhaustion.   1. **Sociocultural Influences**   Women’s preferences, such as preference for female HCPs, could not always be accommodated, sometimes resulting in tension between midwives and women. Some women took traditional herbs before coming to the hospital; these were discouraged as they could lead to complications.  **Women’s preferences**  Not considering women’s preferences compelled some women to deliver at home and healthcare facilities. Miscommunication and lack of information resulted in conflict between women and midwives and harsh treatment.   - **Staff Attitudes**   Midwives tend to have a negative attitude towards women from low socioeconomic status, living with disabilities and young adolescent pregnant women. Midwives reflected that adolescents are supposed to be in school and are at high risk.  The authors concluded that they aimed to gain provider perspective and women towards developing strategies promoting women-centred care while using EBPs. Additionally, the authors conclude that factors that contribute to poor outcomes were revealed (Lusambili *et al.,* 2020:4-14) | **Institutional factors**  **Human resources**  Shortage of human resources also creates challenges as healthcare professionals work long shifts, are overworked, and suffer from burnout. Exhausted healthcare professionals are tense and disheartened, thereby compromising RMC.  **Infrastructure**  The physical space should be in good condition, adequate and partitioned, or the use of screens to ensure privacy and confidentiality.  Effective care requires adequate and functional equipment. |
|  |  |  | **Healthcare professional factors**  **Attributes**  Lack of empathy and sensitivity towards marginalized women.  All women deserve the same kind of RMC without discrimination as women gain trust and good interpersonal relationships ensue. Good communication is essential to avoid misunderstanding.  Aggression towards women may prevail in a work environment where healthcare professionals are overworked, fatigued, and demotivated. |
|  |  |  | **Women related factors**  **Knowledge**  Women need guidance on traditional practices harmful to the mother or baby from HCP. They will be more receptive if they have relevant information.  **Preferences**  Women have varying preferences and expectations. Harmful ones were discouraged, such as ingesting traditional herbs that could have adverse effects on the mother or baby. Cultural beliefs and practices influence women’s choices, which healthcare professionals should familiarize themselves with within their communities. |
| Maung *et al.* 2020  Myanmar | Explored community norms, experiences, and perceptions regarding mistreatment. | This study had both healthcare professionals' and women’s perspectives on disrespect and abuse. The focus will be on the findings of healthcare professionals.   1. **Physical abuse**   Healthcare professionals' views varied on this matter; some indicated that using physical force is unacceptable, while others recounted that sometimes it is necessary to “pinch” or shout at a woman to gain her cooperation.   1. **Denial of care**   HCP reflected that it is unacceptable to deny a woman help; however, it also depends on the type of help, whether possible or not.   1. **Physically restraint**   Restraining is not acceptable for HCP, except if it is to protect a woman from falling, for example, in the case of eclampsia.   1. **Access to resources**   Access to resources entails finances, information, birth companionship, workforce, and physical infrastructure in good conditions.  Financial resources enable the transportation of women to healthcare facilities to seek help; its lack thereof compromises timely access to care. Stable finance and prudent management ensure an adequate supply of medicines, equipment, and medical supplies. The physical space was also limited to accommodate all women. Screens were unavailable to ensure privacy and confidentiality, and medicines and equipment were also in short supply, compromising care.  Lack of access to information can lead to misunderstanding between healthcare professionals and women, especially if they are illiterate. Therefore, women should attend antenatal care early for relevant information about childbirth and birth companionship.  A shortage of healthcare professionals led to an increase in the nurse-patient ratio, which resulted in stress and demotivation related to high workloads. The salaries did not match the work put in.  The authors concluded that there is a dire need to remove any form of disrespect and abuse in healthcare facilities. Furthermore, provider perspectives will contribute to shaping maternity care towards acceptable standards (Maung *et al.,* 2010:5-14). | **Institutional factors**  **Human resources**  Human resources constraints left healthcare professionals overburdened with a high workload, leading to stress, fatigue, and poor maternity care. This kind of environment may lead to aggression towards women.  **Equipment and medical supplies**  Inadequate or unavailability of medicine and equipment compromised care. Financial stability would enable the acquisition of equipment and medical supplies.  **Infrastructure**  Inadequate physical space leads to overcrowding and unnecessary exposure of women. |
|  |  |  | **Healthcare provider factors**  **Attributes**  Empathy and reassurance are comforting for women.  Good interpersonal relationships between HCPs and women result from communicating gently using simpler terms in a language women understand and listening attentively. However, HCP indicated that to get women to cooperate, they had to use ‘force.’ This aspect is neither RMC nor EBP. |
|  |  |  | **Women related factors**  **Knowledge**  Women need health information related to childbirth and companionship to make informed choices. The ability to attend antenatal care allows for the timely identification of any risk factors. Lack of information can result in tense relationships or conflicts between women and healthcare professionals. The information should be simple and in a language the woman understands. |
| Moridi *et al*. 2020.  Iran | Explored the perceptions of Iranian midwives regarding respectful maternity care during labour and childbirth. | The study revealed three themes, each with sub-themes: Showing empathy, Women-centred care, and Protecting rights.   1. **Showing empathy**   Two aspects fell under empathy: establishing friendly relationships and being with women.   - **Establishing friendly relationship**   Most midwives indicated that good interpersonal relations were essential to gain women’s trust. Communicating gently with kindness and compassion leads to good connections and friendships.  Some midwives stated that compassion and allowing a birth companion was essential for respective care.   1. **Women-centred care**   Two sub-themes under women-centred; "keeping women safe and participating in decision making."   - **Keeping women safe**   This meant advocating against unnecessary interventions and the medicalization of childbirth in low-risk women. For this, a midwife-led model is ideal compared to an obstetric model.   - **Participating in decision-making**   Midwives narrated that women should be involved in decision-making and respecting women's preferences and individuality.   1. **Protecting rights**   This theme has three sub-themes; "safeguarding the dignity, giving equal care and preparing the appropriate environment."   - **Safeguarding dignity**   Midwives narrated that women's rights should be upheld and women should be treated with respect and dignity. Mistreatment of women was cited as done mainly by "obstetricians and young midwives" and should be stopped.   - **Giving equal care**   Midwives stated that care rendered to women should be non-discriminatory on any grounds. All women should be treated equally irrespective of background, social standing, or religion.   - **Preparing appropriate environment**   According to the midwives, the birthing environment should be clean, with adequate equipment and space for birth companions.  The authors concluded that RMC is not just about "preventing disrespect and preserving women's rights and dignity" but treating women holistically with compassion. The authors also concluded that there is a need for EBPs, women-centred and humanized care. To achieve this, strategies that promote RMC are a necessity. Policymakers should also be on board in creating a work environment that supports and encourages RMC (Moridi *et al.,* 2020:4-9). | **Equipment and medical supplies**  The birthing environment should have adequate equipment to meet the needs of women for safe and acceptable care. Inadequate supplies compromised RMC.  **Infrastructure**  The birthing environment should be safe and clean to promote security and a sense of belonging for women. There should be adequate space for birth companions to ensure privacy and confidentiality. |
|  |  |  | **Healthcare provider factors**  **Attributes**  Kindness and compassion made women feel acknowledged. Good interpersonal relations with women are essential to gain their trust. Communicating gently and listening to women leads to good interpersonal relations.  **Competence**  Educating healthcare professionals on RMC was important to expedite acceptable care.  Health professionals training on acceptable behaviour and positions to use during childbirth empowered them with the skills needed to promote RMC.  Post-RMC training, HCPs accommodated birth companionship and continuous support despite the challenges.  The ability to advocate against unnecessary interventions and the medicalization of childbirth in low-risk women was vital.  Midwives also acknowledged protecting women’s rights as a basic need for women. |
|  |  |  | **Women related factors**  **Knowledge**  Providing women with health information on childbirth during the antenatal period will empower women.  **Preferences**  HCP accommodated women-centred care by adapting women's choices. "Keeping women safe and participating in decision making." Women-centred care is part of RMC as it empowers women.  They upheld women's rights, so dignity and non-discriminatory care prevailed. |
| Moyer *et al.* 2020.  Ghana | Explored providers' perspectives and behaviour regarding respectful maternity care, including knowledge, attitudes, and practices. | The researchers based their findings on interviews and observations.  **1. Personal definitions of kind and respectful care**  The understanding of RMC varied amongst participants. They ranged from "therapeutic provider-patient communication, proper introduction of yourself as the provider, explanation of procedures and examinations, providing emotional support, allowing patients to deliver in the preferred position, and ensuring privacy and a safe environment for clients.   - **Acknowledgement of disrespect**   Though participants understood RMC, there were instances when the opposite happened. Women were sometimes shouted at or mistreated because they looked 'uneducated or had a high parity'. HCP attributed exhaustion to verbal attacks on women.   - **Behaviours practised and not practised.**   Participants indicated that they explained and gave information to women and allowed them to use preferred positions, though this depended on individual midwives' skills.  As for allowing birth companions, physical space was a challenge for privacy if there was more than one woman in labour.   1. **Birth observations**  - **Communication**   The local language used, and translator when the need arose; lack of communication with women, no explanation of interventions, no greetings nor asked about how they felt by midwives.   - **Privacy**   Physical space posed challenges in providing privacy; women were sometimes exposed unnecessarily; there were no screens between beds in the postnatal ward and overcrowding in the postnatal ward.   - **Verbal and physical abuse**   Women were subjected to all forms of abuse, from physical restraints to physical and verbal abuse.  Differential care  The parity contributed to the care provided; those with high parity were ridiculed.   - **Cleanliness and facility environment**   The environment was kept clean, and principles of sterility were observed, though when the midwives became busy, the trash piled up as they could not keep up with disposal.  The authors concluded that disrespect occurred even from those who meant well. Thus, health professionals need training on RMC to eliminate incidences of disrespect. The training should assist health professionals in handling themselves with compassion and care even under stressful conditions during childbirth (Moyer *et al.* 2020:3-7). | **Institutional factors**  **Infrastructure**  Limited physical space posed challenges such as overcrowding and lack of privacy.  Sometimes, HCPs expose women unnecessarily due to the lack of screens between beds, compromising RMC.  Childbirth support is part of RMC; preferred birth companions were allowed but limited due to inadequate physical space.  The space was limited, causing overcrowding in the labour ward.  Women's mobility was also restricted due to limited space, contradicting the right to mobilise during childbirth. |
|  |  |  | **Healthcare professional factors**  **Attributes**  Compassionate and empathy for women create emotional stability in women. Good communication with women builds a good rapport between women and midwives, but in most instances, midwives do not explain interventions.  Communicating in a language the woman understands to get the message across; using an interpreter is an effective tool for language barriers.  Sometimes, HCPs ignored women and used verbal and physical abuse attributed to stress or to get women to cooperate.  **Competence**  Training provided midwives with knowledge on implementing RMC, though they did not implement it fully. Being selective in the use of EBPs compromises RMC. |
|  |  |  | **Women related factors**  **Knowledge**  For women to understand childbirth, explanation and information was given to women so that they could make informed decisions.  Women have the right to information related to care, explanation and information given to women.  **Preferences**  Women’s preferences were accommodating in line with women-centred care. Preferred birth companions were allowed but limited due to inadequate physical space for more than one woman in labour.  Mobility and preferred positions by women are recommended. Women were allowed to use preferred positions; this was dependent on individual midwife's skills. |
| Mselle *et al.* 2018.  Tanzania | Explored community and skilled health personnel perceptions and practices on humanising birth care. | The findings revealed barriers and facilitators to the provision of RMC.  1. **Barriers**   - **Space and facility limitations**   The participants narrated that the physical space was limited; as a result, women's mobility was restricted and compromised privacy. The participants indicated a need to improve the physical space for respectful and human care.   - **Human resource shortage**   Another issue raised was the large number of women compared to midwives, resulting in an increased workload that compromised care.   - **Institution norms and practices**   Participants said family members were not involved in the care as they were sent home after dropping off the women, only to come during visiting times.  Beliefs that the birth position should be limited to the preferred position by midwives - the lithotomy, as it was convenient for them; women did not choose the birthing position as midwives dictated.   - **Preference of EBP over traditions and culture**   According to midwives, Western practices were preferred over women's cultural traditions. To safeguard the unborn baby's health, midwives discouraged women from using traditional herbs that could potentially have adverse effects on the foetus.   1. **Facilitators**   The study revealed several facilitators of RMC. Participants indicated that there were aspects that could expedite RMC.   - **Ongoing education of HCPs on respectful maternal care**   The study revealed that the HCP had received training on acceptable behaviour and positions during childbirth. Institutional norms designed for continuous clinic support during childbirth.  Participants indicated they did their best to accommodate birth companionship and continuous support.   - **Belief in the benefits of having family members present during childbirth.**   Women were allowed to have family members when it was possible to accommodate them within the limited space.   - **Respecting maternal wishes when appropriate**   Accommodating women's wishes depended on the implication for maternal and foetal wellbeing. Also, some traditional practices were accommodated, such as taking the placenta home by the family.  The authors concluded that despite the barriers, there are also facilitators of RMC that can be heightened in health facilities. However, staffing norms and physical structure challenges need to be addressed to do so. The authors also concluded in-service RMC should be an integral part of maternity care (Mselle *et al.,* 2018:5-9). | **Institutional factors**  **Human resources**  Increased workload due to shortage leads to stress, fatigue and inability to provide one-to-one care.  **Infrastructure**  Adequate physical space was essential, but the area was limited, and as a result, women's mobility was restricted, and privacy was compromised.  The physical space needed to improve to enable respectful and human care. |
|  |  |  | **Healthcare professional factors**  **Competence**  Training for midwives is needed to improve skills and be relevant through training.  Midwives needed support and empowerment through training to provide EBP care.  A supportive environment and teamwork are essential for a healthy working environment for encouragement and motivation, but collegial support was absent due to exhaustion-related work overload. Demotivation contributes to poor maternity care. |
|  |  |  | **Women related factors**  **Knowledge**  Women should receive relevant information during the antenatal period and discuss their birth plans to establish expectations before childbirth.  **Preferences**  HCPs should incorporate EBPs with the woman's preferences.  Unhealthy traditional habits, such as herbs that had adverse effects on the foetus, were discouraged.  Cultural sensitivity was practised as some traditional practices, such as taking the placenta home by the family, were accommodated. |
| Ndwiga *et al.* 2017.  Kenya | Explored provider perspectives on respectful maternity care | An intervention package for RMC was introduced but without a comparison group. Pre-intervention data was collected from "September 2011 and February 2012" and post-intervention data "between January and February 2014." The interventions included "health care providers and their managers to improve providers' attitudes, working environments, facility management, and links to communities."  The study revealed three main themes on factors influencing maternity care after implementing an intervention package for RMC.  **1**. **Understanding and operationalisation of client's rights**  The findings after the intervention were that many health professionals involved women in decision-making by providing them with information about their care.  Aspects such as privacy, and informed consent also improved at the "end line."  However, despite the availability of items such as screens to maintain privacy, midwives narrated that the staff shortage was still a hindrance to providing optimal care.  **2**. **Providers' work-related environment**  The work environment has an impact on the emotional well-being of the midwife. Though interpersonal relations improved, the exhaustion and fatigue from overworking remained unchanged after the intervention.  **3.** **Perceptions on supervision, job fairness and management**  Though participants reported improved leadership, they were still dissatisfied with job fairness linked to high workload and limited infrastructure.  Improvement in teamwork, communication and interpersonal relations with both patients and colleagues after training. However, poor salaries, lack of promotion opportunities, and poor working conditions were demotivating. Others indicated that incentives for work done would be a motivating factor.  The authors concluded that to succeed in providing RMC, there is a need for a mind shift for care providers. Intervention packages that assist in behavioural change and practise should be implemented regularly. However, the work environment should also be conducive to mitigating burnout, which negatively affects patient care. Thus, providers need support to achieve RMC (Ndwiga *et al.,* 2017:7-13). | **Institutional factors**  **Human resources**  Increased workload due to shortage compromised maternity care due to high workload, stress, and demotivation. Healthcare professionals were still dissatisfied about the injustice linked to a high workload.  HCP needs favourable working conditions.  Supervision and job recognition through promotion and incentives motivate healthcare providers towards providing RMC. Poor work environment and low salaries are demotivating.  Teamwork is good as it promotes collegial support.  **Equipment and medical supplies**  Functioning equipment and adequate general supplies are essential to ensure optimal maternity care; its lack thereof compromises patient care. Post RMC training management screens to mitigate and maintain privacy.  **Infrastructure**  Lack of adequate physical space compromised patient privacy as it led to overcrowding. |
|  |  |  | **Institutional factors**  **Human resources**  Demotivating aspects were poor salaries, lack of promotion opportunities, lack of incentives, support from superiors and poor working conditions were demotivating and affecting quality maternity care. Supervision should be fair and consistent.  **Healthcare professional factors**  **Attributes**  Interpersonal relations with women improved.  Training of RMC improved healthcare professionals’ attitudes towards women.  Many health professionals involved women in decisions through information sharing about their care. |
|  |  |  | **Women related factors**  **Knowledge**  The right to information was exercised by involving women in decision-making by providing information about their care post-intervention. The information allows women to make autonomous decision-making.  **Preferences**  Women-centred care entails considering women's choices during the provision of care, of which women’s preferences were deemed. |
| Oluoch‑Aridi *et al.*  2018  Kenya | Explored healthcare providers’ experiences and perceptions of mistreatment during childbirth. | The study’s findings comprised women and healthcare professionals. For this study, only those about the delivery of care by healthcare professionals will be of focus.   1. **Health systems conditions and constraints**   According to healthcare professionals’ accounts, the poor state of the physical structure, inadequate space, lack of water and even electricity, and lack of equipment, beds and medical supplies contributed to the poor quality of care received by women.  Healthcare professionals at referral hospitals cited the high number of referrals compared to the healthcare professionals and very “difficult-to-manage” women, which led to stress and bad temperament. Healthcare professionals manage many women at once, resulting in fatigue and burnout.   1. **Culture of blame**   HCP complaints reflected blame by superiors for adverse outcomes, which drove them to be ‘aggressive’ in providing care as a preventative measure. There was a lack of support from the managers.  The authors concluded discrimination and mistreatment of women were worsened by the disparity in the socio-economic status of healthcare professionals and the communities they served. Women with poor social standing were mistreated. The authors further concluded that there is a need for collaboration between health institutions and professional regulating bodies to hold those who violate women’s rights accountable (Oluoch‑Aridi *et al.,* 2018:5-13). | **Institutional factors**  **Human resources**  Increased workload due to shortage compromised maternity care due to high workload, stress, and demotivation.  Lack of support from supervisors demotivating  Blame rather than remedial and debriefing for adverse outcomes.  **Equipment and medical supplies**  Inadequate medical supplies and equipment compromised RMC.  **Infrastructure**  Lack of adequate physical space compromised patient privacy as it led to overcrowding. The dilapidated building was dehumanizing. |
|  |  |  | **Healthcare professional factors**  **Attributes**  Burnout and stress lead to poor interpersonal relations with women. Sometimes, verbal and physical abuse was inflicted on women. |
| Smith *et al.* 2020.  Zambia | Explored barriers to provision of respectful maternity. | The study revealed five barriers to the provision of respectful maternity care.  **Barrier 1. Providers do not consider the decision to provide respectful care because they already believe they are providing it.**  The participants indicated that they were performing within the norm.  Training, supervision, and feedback focus on clinical treatment and health risks and do not address respectful care.  The focus is on clinical algorithms and guidelines, including visual cues in the facility, but nothing provides clear guidelines for giving good care.  Pain was seen as a natural birth experience — the provider had a painful delivery and has attended many painful deliveries, and the bible says that labour is painful.  Based on experience, some participants stated that intuitively, they knew what to expect from women and the care to provide.  **Barrier 2. Normalizing abuse and violence**  Some HCPs narrated that they received violence as children, so violence was a 'norm' as it enforced discipline.  Training and clinical experience of providers reinforce that clients need rigid, forcefully delivered commands and interventions.  Participants indicated that 'force' was sometimes necessary to prevent complications.  **Barrier 3. Respectful care is not a necessity.**  There were no serious consequences for providers who engaged in disrespectful or abusive behaviour. Providers indicated no consequences for unacceptable behaviour, so nothing deterred them.  Client clothing or appearance makes them seem low income, or they are considered a community member of an inferior status.  Well-dressed women were favoured.   - **Lack of engagement between HCP and woman before childbirth**   The lack of an interpersonal relationship established during the antenatal period can contribute to anxiety in labouring women as they meet for the first-time during labour.  **Barrier 4. The costs of providing RMC outweigh the gains.**  Maternal or infant death results in an audit, emphasising clinical practice.  Clinical audit provides a tool for 'self-awareness and improvement of practices.  Providers do not receive salient information or feedback on the impact of respectful or disrespectful care on health outcomes.  It was evident that midwives’s knowledge of respectful maternity was limited.  **Barrier 5. The use of force will make the woman co-operate.**  In certain instances, midwives used force to have the patient co-operate.  The authors concluded that women's experiences and outcomes should be positive. Therefore, there is a need to implement interventions that uphold RMC (Smith *et al.* 2020:4-10). | **Institutional factors**  **Human resources**  Lack of support contributed to practices that are not evidence-based.  Lack of feedback mechanism on the impact of respectful or disrespectful outcomes |
|  |  |  | **Healthcare provider factors**  **Interpersonal relationships**  Discriminatory care: care for women was based on appearance; those that looked good were treated with respect.  Lack of engagement with the woman before childbirth contributed to anxiety in labouring women.  In certain instances, midwives used force as a way of getting the patient to cooperate.  Lack of proper understanding of RMC by midwives resulted in ineffective implementation.  Beliefs influence care, e.g., labour pains are viewed as 'natural', so analgesia is unnecessary.  The clinical experience influenced the care provided, so force was justified as necessary sometimes to prevent complications. |
|  |  |  | **Women related factors**  **Knowledge**  Women lacked knowledge, so midwives decided for women instead of providing information.  Midwives believed they knew what was best for the woman, though the care was not women-centred. |
| Warren *et al.,* 2017.  Kenya | Described mistreatment of women in Kenya. | The study is part of a larger project from the Heshima project, a participatory implementation research study in Kenya.  HCP perspectives on health system conditions and constraints and drivers of D&A were used in the discussion.   1. **Health system conditions and constraints**  - **Facility culture**   According to HCP, lack of answerability by management for infrastructure maintenance, a dirty environment, inadequate equipment, medical supplies, high patient-provider ratio, and lack of supervision contributed to poor quality care.   1. **Drivers of disrespect and abuse during childbirth**  - **Health system-level drivers**   Various factors contributed to disrespect. “Governance and leadership, service delivery and healthcare providers.”   - **Governance and leadership**   Poor management contributed to poor infrastructure shortage of HCP and consequentially high workload resulting in burnout, stress, and demotivation. Consequently, burnout compromised patient care.   - **National laws and policy-level drivers**   The lack of consequences for neglect of women perpetuates the mistreatment of women, compounded by a lack of training on human rights. HCPs are not equipped with basic skills to uphold human rights.  The authors conclude that there is a need to address disrespect and abuse drivers as evidence keeps emerging. Through identifying drivers of D&A, specific strategies can be implemented to address the issue Warren *et al.,* 2017:8-13). | **Institutional**  **Human resources**  Adequate human resources contribute to quality maternity care as one-to-one care is visible. When inadequate healthcare professionals are overworked, stressed, have burnout, and are demoralized, compromising RMC.  Lack of redress for disrespectful care perpetuates the disrespect.  Ongoing supervision and job performance acknowledgement would give HCPs a sense of appreciation and belonging.  **Equipment and medical supplies**  Inadequate equipment and medical supplies contribute to substandard care and D&A.  HCPs are frustrated due to a lack of essential medical supplies and equipment, as they cannot perform their duties effectively.  **Infrastructure**  Physical infrastructure should be well maintained and adequate to prevent overcrowding and compromising privacy. |
|  |  |  | **Healthcare professional factors**  **Attributes**  To ensure sustained good interpersonal relationships, midwives need to communicate gently and attend to the needs of women. Harshness is not representative of RMC.  **Competence**  To provide RMC, healthcare professionals need to be equipped with the related information. Lack of training on RMC and human rights contributes to the D&A of women. |

**Additional file 5**: **Healthcare institution-related factors**

| **Study** | **Human resources** | **Medical resources** | **Norms and practices** | **Physical infrastructure** |
| --- | --- | --- | --- | --- |
| Afulani et al. 2020  Kenya | - Shortage of healthcare professionals resulted in an increased workload, physical and mental fatigue, and poor work output.   *“Staff shortage is a big issue; you can find yourself on night duty at the same time you are covering daytime, and so you can’t get good services that you want to give a client because you are exhausted.”* | - Inadequate medical supplies, equipment and drugs compromised the quality of care, leading to frustration. | - Lack of accountability and redress perpetuated a culture of D&A. Mistreatment of women was normalised without any consequences. | - The physical space was inadequate and compromised privacy and confidentiality.   “…*we have to treat them as individual and give them their privacy. But also because of the space that is available, and we have to help all these clients, it forces you to mix them, and also as you speak to this one, the other one will also have to hear what you are saying to this one.”* |
| Asefa et al. 2020.  Ethiopia | - Shortage of midwives leads to increased workload, long working hours, and poor work output, compromising patient care.   *“Practicing midwifery is difficult in Ethiopian facilities; as a midwife, you are supposed to work for 24 hours if you have night duty, the workload is exceptionally high, you take care of several women at a*  *time. Disproportionately, your salary is very low, as is your risk payment.”* | - Inadequate screens meant no privacy for women, compromising respectful care.   *“Because we do not have adequate [privacy] screens, we are asking all labour companions to go out of the ward whenever a woman is to have an examination…”*   - The health professionals demanded constant availability of services, resources, and adequate stock of supplies to ensure quality care. | - The lack of incentives and recognition of HCPs was demotivating, resulting in compromised patient care.   *“Sometimes, you keep on doing and discharging your responsibilities appropriately, but no one from the senior managers comes to you and*  *“For example, I had a long night assisting women, but I am not paid fairly. Is it fair to accuse me of violating women’s rights? I do not think… a lot must be done from top to bottom in responding to providers’ right before trying to maintain women’s right.”*  *“…in 99% of the cases, health facility management attend only to the rights of clients; they do not emphasise the rights of care providers…”* | - The health professionals demanded refurbishing dilapidated physical structures to ensure adequate space for privacy. |
| Dzomeku et al. 2020a.  Ghana | - Lack of redress compromised RMC. | - ___ |  | - ___ |
| Dzomeku et al. 2020b.  Ghana | - A shortage of midwives leads to increased workload, long working hours, and poor work output, compromising patient care. - Participants expressed the need for more staff and improvement in salary scale. | - Inadequate supplies such as screens were insufficient to provide privacy, compromising respectful care.   *“With the ward, providing privacy is one of our biggest challenges…we have fewer screens here. Our screens are not many. You may be using a screen for a patient, the other one too may need a screen but they both can’t have it at the same time so one has to be exposed whilst the other one is screened. So, if the screens are many, and I think each cubicle should have the screen so that the patient will have their privacy.”*   - The health professionals demanded constant availability of services, resources, and adequate stock of supplies to ensure quality care. | - Discriminatory care prevailed.   *“It is because the mother is infected with HIV that is the reason why my colleagues didn’t want to treat her...”* | - The health professionals demanded refurbishing dilapidated physical structures to ensure adequate space for privacy. |
| Lusambili et al. 2020 Kenya | - The shortage of HCPs leads to high workloads and burnout.   *“Shortage of staff it comes in, suppose the person is alone there are many active cases, now which also contributes, there are burnouts…”* | - Adequate screens are a necessity in ensuring privacy.   *“The first thing, if we can get enough equipment which is needed in the maternity. Secondly, we need like there’s supposed to be a screen when a mother is in the delivery couch even the cleaner should not see that mother…”* |  | - The physical was inadequate to accommodate all the women individually.   *“… for privacy we need, we’ll need space, which is very important, because we have very many mothers who actually need service. We have even the doctors, they don’t have anywhere to do their examination, they are using the same couch; so, I think the most important thing to be done here in xxx hospital is space, we need space for that…”* |
| Maung et al. 2020  Myanmar | - Human resources constraints left healthcare professionals overburdened with a high workload, leading to stress, fatigue, and poor maternity care. This kind of environment may lead to aggression towards women. - *“Much work load. At first, there were only three doctors here. It was not balance. We had to take night duty successively. So, when it happened many days, we became tired and were not in good mood.”* | - Lack of screens compromised privacy. - Necessities such as medicine and equipment were in short supply, compromising care.   *“The Government has been providing medicines. But, when we are actually in need, we have run out of medicine.”* | - ___ | - Inadequate physical space leads to overcrowding. - *“If we allow an outsider (a patient attendant) to come in, we have to worry about infection. In the present condition, we allow patient attendants to come into the waiting room, not into the delivery room.”* |
| Moridi et al., 2020.  Iran | - ____ | - The birthing environment should have adequate equipment to meet the needs of women for safe and acceptable care. Inadequate supplies compromised RMC. |  | - The birthing environment should be safe and clean to promote security and a sense of belonging for women. Adequate space for birth companions to ensure privacy and confidentiality. - *“The companions stay outside in a cold place, from night until morning. They lose their patience, so they may get easily nervous. If they could stay in a suitable place, they would be comfortable and cooperate with us to support the women.”* - *"When a woman enters the birth unit and sees an untidy environment, she may get stressed and feel some sort of disrespect; we should try to provide a convenient setting in which women could be relaxed.”* |
| Moyer et al. 2020.  Ghana | - Increased workload due to shortage leads to stress, fatigue, and inability to provide efficient care. | - ___ |  | - Limited physical space posed challenges such as overcrowding and lack of privacy. - There was more than one woman in labour, causing overcrowding.   *“Dignity means to preserve the privacy of women without unnecessary exposure. There is not much privacy…if we are monitoring two women simultaneously; we will just explain to the people that their relatives cannot enter because there are two women. So, with the health staff, we just monitor them.”*   - Women's mobility was also restricted due to limited space, contradicting the right to mobilise during childbirth. |
| Mselle et al., 2018.  Tanzania | - Increased workload due to shortage leads to stress, fatigue, and inability to provide one-to-one care. | - ___ |  | - Adequate physical space was essential, but the area was limited; as a result, women's mobility was restricted and compromised privacy. - The physical space needed to improve to enable respectful and human care. - *"It is impossible [for mothers to walk around in the labour room] given limited space in our labour room. The room has a little space for a bed and a small table."* |
| Ndwiga et al., 2017  Tanzania | - Increased workload due to shortage compromised maternity care due to high workload, stress, and demotivation.   *“…sometimes nurses experience burnout related to high workload, sometimes related to outcomes like a maternal death. And it would put them in a situation where they are not able to cope.”* | - Functioning equipment and adequate general supplies are essential to ensure optimal maternity care; its lack thereof compromises patient care. Post-RMC training, management provided screens to mitigate and maintain privacy. |  | - Lack of adequate physical space compromised privacy for patients as it leads to overcrowding. |
| Oluoch‑Aridi et al.  2018  Kenya | - Increased workload due to shortage compromised maternity care due to high workload, stress and demotivation. | - Inadequate medical supplies and equipment compromised RMC. |  | - Lack of adequate physical space compromised patient privacy as it led to overcrowding. The dilapidated building was dehumanizing. |
| Smith et al. 2020.  Zambia | - ____ | - ____ | - The welfare of HCP is not considered.   “*The focus is usually on clinical algorithms and guidelines, including visual cues that do not provide clear guidelines for “good care” at organisational level.”* | - ___ |
| Warren et al.  2017.  Kenya | - Adequate human resources contribute to quality maternity care as one-to-one care is visible. - Inadequate and overworked HCPs have burnout and demoralised, compromising RMC. To which ongoing support would have provided a sense of appreciation. - Lack of redress for disrespectful care perpetuates the disrespect | - Inadequate equipment and medical supplies contribute to substandard care and D&A. - Healthcare professionals are frustrated due to a lack of essential medical supplies and equipment, as they cannot perform their duties effectively. |  | - Physical infrastructure should be well maintained and adequate to prevent overcrowding and compromising privacy. |

**Additional file 6: Healthcare professional-related factors**

| **Study** | **Attributes** | **Competence** |
| --- | --- | --- |
| Afulani *et al.* 2020.  Kenya | - Inability to cope with workload lead to bad temperament resulting in uncaring behaviours towards women.   *“Maybe it is the staff’s attitude, if also the staff cannot control her stress, she projects] her stress to the clients.”*   - Discriminatory care for women sometimes prevailed.   *“Some is just physical appearance; you just get in, and everybody is in love with her, and the other one comes in, and everyone is like oooh [laughs] nobody bothers to attend to her, but mostly it is race and financial status.”* | - Training on RMC for midwives was a need to acquire competencies to provide RMC.   *“…training would be useful on] how to handle patients who are hostile and who cannot cooperate…”*  *“I think that continuous education will help stop this discrimination issue…”* |
| Asefa *et al.* 2020.  Ethiopia | - Healthcare providers were sensitive to women’s needs. - Effective communication with women and communities is vital to avoid misunderstandings and ensure sustained relationships. - D&A was attributed to uncooperative behaviour by the woman.   *“It [verbal abuse] happens on a few cases where the mother is refusing to cooperate during delivery. . .We are not doing this to harm the mother but to save the life of the baby.”* | - Training on RMC provided participants with more insight. They became more aware, reflected on their own behaviour prior to training, and corrected undignified practices. "Role play, case scenarios, video shows and participatory."   *“…we used to provide care with force if women refused to have a procedure or an examination. After the training, there is no such practice; we provide services with consent and respect.”* |
| Dzomeku *et al.* 2020a.  Ghana | - Midwifery was a “calling,” not just any other job. Empathy and sensitivity were a need. - Good interpersonal relations are a necessity in ensuring respectful care. - Healthcare professionals acknowledged the importance of upholding women’s rights, women-centred care, and non-abusive care as a representation of RMC.   *“You must show love and welcome her, so she feels she has come home. In that way, even if she came with some anxiety, she will become relaxed.”*   - Hitting women’s thighs during "bearing down" to ‘encourage’ cooperation.   *“Well, it’s bad to hit childbearing women I only [do it] when conducting delivery and she is closing the gap, and I hit in-between the thighs ‘open up!’, aha, that’s the only time I hit a childbearing woman, and it is not hitting deliberately. “* | - Midwives were aware of the components of RMC: informed consent, privacy, and confidentiality, having a companion of choice and health information, and client-centred, non-abusive care, which is a positive indicator of the provision of RMC.   *“Non-abusive care is the care you give to childbearing women without using abusive words, like insults or shouting or using non-verbal languages to communicate with childbearing women.”*  *“You [the midwife] are there to support that childbearing woman at that moment, so, you assist in sacral massage, and you give her encouraging words in order that the childbearing woman will not feel that she is in that alone. There are a lot of childbearing women in there, so we are in as their support to go through their labour and the pain…So, we are the caregivers, and we are also the supporters . . . we are giving them the support during the labour.”* |
| Dzomeku *et al.* 2020b.  Ghana | - Exhaustion and stress due to a high workload may have led to aggression towards women and poor interpersonal relationships. - *“If it comes to the attention that you are just a petty trader in the market, to put it mildly, some of these petty traders are not exceptionally neat, not their fault, but a lot are unkempt. So, when they are coming to labour, instead of taking a bath, shave, do the necessary little stuff that makes a woman presentable, she just picks a bag and presents herself to the ward. Sometimes, you open that bag, and it is full of bed bugs. So, if you don’t hold yourself in check, you will get angry.”*   *“Oh, it happens all the time. The hitting, it is an everyday occurrence…even you [the interviewer], they [midwives] will insult you when you come here. Who are you?”* | - Care should have a scientific basis, but the abuse was sometimes justified to get women to cooperate and follow commands, although the force had no scientific basis. - *“She told me the baby is coming, so I told her to lie on the floor because if she stands, the baby can hit the floor. Therefore, I told her to lie on the floor. However, this lady didn’t do it but rather, how do I do it, but rather, I don’t even know how to say it, she squatted or something and in Ghana here, or in this hospital, the patient, you are supposed to lie on your back. Therefore, she was squatting. I told her to lie on the back. Moreover, she was like, ‘No, this is what I want’. In addition, I told her, ‘You cannot do this to deliver, please, lie on your back’. So, I held her hand, and I turned her to lie on her back, but this woman refused to open the thigh for me to even do the delivery.”* |
| Lusambili *et al*. 2020 Kenya | - Lack of empathy and sensitivity towards marginalised women. - All women deserve the same kind of RMC without discrimination to ensure trust and good interpersonal relationships. - Good communication is essential to avoid misunderstanding. | - ___ |
| Maung *et al.* 2020.  Myanmar | - Empathy and reassurance are comforting for women. - Good interpersonal relationships exist when healthcare professionals communicate with empathy. However, to get women to cooperate, midwives reflected on the need to use ‘force.’ This aspect is neither RMC nor EBP | - ___ |
| Moridi *et al*. 2020.  Iran | - Kindness and compassion made women feel acknowledged. - Birth companionship and continuous support were accommodated despite the challenges. - Good communication with women is essential to gain their trust.   *“We respect the women when we give information about the progress of labour. We should tell them what is going on at every stage and what they can do to help themselves; this would reduce their stress. We should introduce caregivers and make them familiar with rooms and equipment as well.”* | - Health professionals training on RMC empowered HCPs with the skills needed to promote RMC.   *First, we all want to give scientific care and perform it accurately. We don't need to do some interventions such as unnecessary vaginal examinations, forced early admission and electronic foetal heart monitoring continuously during labour and birth.”*   - The ability to advocate against unnecessary interventions and the medicalisation of childbirth in low-risk women was not always possible.   *“Disrespect is a consequence of working in a medicalised context. They (obstetricians) treat labouring women hastily and completely medically. If we had midwife-led birth centres, then we could provide respectful care for women.”* |
| Moyer *et al.* 2020.  Ghana | - Good communication with women creates a good rapport between women and midwives, but in most instances, no explanation of interventions was given. - Communicating in a language the woman understands gets the message across; a translator was used to avoid barriers to effective communication. - Sometimes, verbal, and physical abuse was inflicted, attributed to stress or to get women to cooperate.   *“Respectful maternal care it is giving the woman the right care. Making her to feel like she is in her own local setting. So that, psychologically, physically, and mentally, she is okay throughout the process she has made or the services you are rendering to her. The woman should feel like she is at home and appreciate the services afterwards.”* | - Training provided midwives with knowledge on implementing RMC, though they did not fully implement it. Selective in the use of EBPs compromises RMC.   *Sometimes we get so overwhelmed…there are times we are not able to explain the procedure to the patient as we are supposed to … there are times that we just focus on getting the results, getting the tasks done, and not really remembering to work with the patient.”*   - Basic education and training were the foundation for knowledge and skills.   *“We have learned in books how contractions can make women misbehave. Moreover, some of us have gone through labour, so we know how it is. So, we put ourselves in their shoes and just handle them as gently as possible.”* |
| Mselle *et al.* 2018.  Tanzania | - Support persons provide comfort to women during childbirth, but no support persons, relatives or friends were allowed. - Visitors were only allowed in the ward during visiting times. | - Training and practice sharpened skills for midwives.   *“Once you learn for the first time, you don't forget how to cook …You already know that I will do this, I will do this, I will do this. As we were taught, we have to allow the woman to assume the position in which she is comfortable to give birth….”*  *“You are [midwife] the one who tells the woman which position she should lie for easy delivery because other women, it is their first delivery, so they do not know”.*   - Midwives need support and empowerment through training to provide EBP care.   *“(…) we were educated about the importance of maintaining privacy, ensuring the woman in labour is constantly supported physically and emotionally by providing psychological support and managing pain (…)”.* |
| Ndwiga *et al.* 2017  Tanzania | - Interpersonal relations with women improved after training on RMC. - Women were involved in decision-making.   *“The element of communication improved; midwives could communicate very well with clients, and it reduced some of the clients' perceptions of disrespect and abuse … another thing which worked very well was teamwork, which started at the site of implementation; the maternity itself, the nursing office... right to the health management team.”* | - ___ |
| Oluoch‑Aridi *et al.* 2018  Kenya | - Sometimes, HCPs inflict verbal and physical abuse on women.   *“…You know some mothers are uncooperative because of the pain you find a woman if you are so gentle with them, they become unruly, but if you are so gentle and so, you can find some of them even injuring the baby, so you have to be ‘firm’…”* | - ___ |
| Smith *et al.* 2020.  Zambia | - Force was justified as necessary sometimes to prevent complications.   “The most important thing is to deliver a live baby. When they go into second stage, you must be very vigilant.”   - Discriminatory care: care for women was based on appearance, such as nice clothes and good hygiene. - Midwives’ beliefs influenced care, e.g., labour pains were viewed as 'natural', so analgesia was unnecessary.   *“…painful deliveries are the norm, and scripture and religious messaging on the pain inherent to childbirth.”* | - ___ |
| Warren *et al.* 2017.  Kenya | - To ensure sustained good interpersonal relationships, midwives need to be sensitive to the needs of women. Stress and burnout compromise RMC. - Physical abuse was justified.   *“The issue of mothers saying nurses are bad must be addressed in the community. They should be informed that nurses are not bad. Ideally, any mother who has delivered in a hospital is slapped on the thighs to facilitate or encourage her to push because if the mothers do not push the danger is obvious.”* | - To provide RMC, healthcare professionals need to be equipped with the related information. Lack of training on RMC and human rights contributes to the D&A of women. |

**Additional file 7: Women related factors.**

| **Study** | **Knowledge** | **Preferences** |
| --- | --- | --- |
| Afulani et al. 2020  Kenya | - ___ | - **___** |
| Asefa et al. 2020.  Ethiopia | - Women need health information about pregnancy and childbirth during the antenatal period to make informed decisions. - Similarly, the role of the support person needs clarification during the antenatal period to avoid role confusion between the support person and the midwife. - Families and communities need to be on board through health education as well. | - ___ |
| Dzomeku et al. 2020a.  Ghana | - Lack of knowledge by women of childbirth compromises women’s ability to exercise autonomy. - *“The right to care*, *the right to information*, *the right to privacy*, *the right to the best of care and the right to consent to any procedure supposed to be performed on her*, *and the right to get an explanation concerning any procedure supposed to be performed on her.”* - During the antenatal period, women should receive information in preparation for childbirth to allow involvement in decision-making. - *“…from antenatal, she is knowing that this is how labour is, this is how when the baby is about coming, this is how it is, this is how you have to attach your baby to the breast, “even we ourselves, we won't get tired/frustrated” because she knows so she will act according to that information she is having.”* | - Women should exercise the right to choose.   *She also has that right. And then, for instance, a childbearing woman can ask for an alternate, say an oral medication instead of an injection, because she may not like injections. For that, too, she has a right because she can’t take an injection, and as long as an alternative exists that she can orally ingest, she has that right. Also, any information concerning her care, she has every right to have access to it. . .* |
| Dzomeku et al. 2020b.  Ghana | - ___ | - Women need information about childbirth and what is expected to cooperate and practice autonomy. |
| Lusambili et al. 2020 Kenya | - Women must receive health information about traditional practices harmful to the mother or baby.   *“… Okay, me what I can say about the cultural beliefs what is supposed to be done? These people need to be educated. About the cultural beliefs, about the norms, because you cannot encourage them to continue practicing, to continue practicing the, to continue with their norms. So, if it’s delivery, if it’s delivery but not herbs, me what I can say in delivery …”* | - Women have varying preferences and expectations. Harmful practices that could adversely affect either the mother or baby were discouraged.   *“ ..Yes, there are beliefs in the community, but for example, there is a belief issue of substitution. For example, if they eat eggs, they will be very fat, and there are religions which don’t allow people to deliver in a health facility...”*   - Cultural beliefs and practices influence women’s choices.   *“What I think about it, she might need this man, the woman, to deliver her, but in the real sense, the female staff is not there. So, this one, as I have said, they need to be health educated, to be, those about the, about the cultures.”* |
| Maung et al. 2020  Myanmar | - Women need information by attending antenatal care; they receive health information related to childbirth and companionship to make informed choices. The ability to participate in antenatal care allows for the timely identification of risk factors. Lack of information can result in tense relationships or conflicts between women and healthcare professionals. The information should be simple and in a language the woman understands. | - ___ |
| Moridi et al. 2020.  Iran | - Providing women with health information on childbirth during the antenatal period will empower women.   *“When they come here, the rights of women from every culture and tradition should be protected, and we must pay attention to them.”* | - Women-centred care is part of RMC as it empowers women; thus, HCP accommodates women’s preferences. *"Keeping women safe and participating in decision making."* - To ensure dignity and non-discriminatory care, HCP upheld women's rights. |
| Moyer et al. 2020.  Ghana | - For women to understand childbirth, explanation and information was given to women so that they could make informed decisions.   *“If she is conscious, we can explain to her as well as to the relatives. But if she is not conscious, we can tell the relatives about the condition. Any time we are doing it, we explain to them.”* | - Women’s preferences were accommodating in line with women-centred care. Preferred birth companions were allowed but limited due to inadequate physical space for more than one woman in labour. - Women were allowed to use preferred positions, though this was dependent on individual midwife's preference. |
| Mselle et al. 2018.  Tanzania | - Women should receive relevant information during the antenatal period and discuss their birth plans to establish expectations before childbirth. | - A woman's preferences should be accommodated where possible.   *“…although most of the time, we advise them to lie on their back. But if she wants to squat, you have to allow her.”*   - Unhealthy traditional habits, such as herbs that had adverse effects on the foetus, were discouraged. - Cultural sensitivity was practised as some traditional practices, such as taking the placenta home by the family, were accommodated. - *“In my perspective, we are not that thoughtful of the traditional practices, but we are always accommodative of the mother and family wishes if there are any. Whether it's the longing to pack up the placenta back home or this and that, one should simply let the mother fulfil her wish.”* |
| Ndwiga et al. 2017  Tanzania | - The right to information was implemented by involving women in decision-making by providing information about their care post-intervention. - Information allows women to make autonomous decision-making. | - Women-centred care entails considering women's choices during the provision of care, of which women’s preferences were deemed.   *“You know, in our setup, the client is always right, so if they say I don’t want to be in this position, or I do not want to stand here for long, then you explain to the client the risks and benefits then the client makes the decision. ”* |
| Oluoch‑Aridi et al. 2018  Kenya | - __ | - *“One of the best supports that we can provide for a woman is to allow a companion of her choice to stay with her. If the companion has taken part in the birth-preparation classes, she or/he can help the mother to have a better experience.”* |
| Smith et al. 2020.  Zambia | - Women lacked knowledge, so midwives made decisions on behalf of women. - Midwives believed they knew what was best for the woman, so women-centred care was not practised. | - ___ |
| Warren et al. 2017.  Kenya | - ___ | - ___ |
